# NEDD4L intramolecular interactions regulate its auto and substrate Na_V_1.5 ubiquitination

**DOI:** 10.1016/j.jbc.2024.105715

**Published:** 2024-02-02

**Authors:** Katharine M. Wright, Sara Nathan, Hanjie Jiang, Wendy Xia, HyoJeon Kim, Nourdine Chakouri, Justin N. Nwafor, Lucile Fossier, Lakshmi Srinivasan, Zan Chen, Tatiana Boronina, Jeremy Post, Suman Paul, Robert N. Cole, Manu Ben-Johny, Philip A. Cole, Sandra B. Gabelli

**Affiliations:** 1 Department of Biophysics and Biophysical Chemistry, The Johns Hopkins School of Medicine, Baltimore, Maryland, USA; 2 Division of Genetics, Department of Medicine, Brigham and Women’s Hospital, Boston, Massachusetts, USA; 3 Department of Biological Chemistry and Molecular Pharmacology, Harvard Medical School, Boston, Massachusetts, USA; 4 Department of Pharmacology and Molecular Sciences, Johns Hopkins School of Medicine, Baltimore, Maryland, USA; 5 Department of Physiology and Cellular Biophysics, Columbia University, New York, New York, USA; 6 Mass Spectrometry and Proteomics Facility, Department of Biological Chemistry, Johns Hopkins University School of Medicine, Baltimore, Maryland, USA; 7 Department of Oncology, The Johns Hopkins University School of Medicine, Baltimore, Maryland, USA; 8 Department of Medicine, The Johns Hopkins University School of Medicine, Baltimore, Maryland, USA

**Keywords:** NEDD4L, NEDD4-2, HECT, E3 ligases, ubiquitin, Nav1.5, SCN5A, voltage-gated sodium channel, post translational modification, PTM, transthioesterification, proteostasis, mass spectrometry, nanobody, NanoMaN

## Abstract

NEDD4L is a HECT-type E3 ligase that catalyzes the addition of ubiquitin to intracellular substrates such as the cardiac voltage-gated sodium channel, Na_V_1.5. The intramolecular interactions of NEDD4L regulate its enzymatic activity which is essential for proteostasis. For Na_V_1.5, this process is critical as alterations in Na^+^ current is involved in cardiac diseases including arrhythmias and heart failure. In this study, we perform extensive biochemical and functional analyses that implicate the C2 domain and the first WW-linker (1,2-linker) in the autoregulatory mechanism of NEDD4L. Through *in vitro* and electrophysiological experiments, the NEDD4L 1,2-linker was determined to be important in substrate ubiquitination of Na_V_1.5. We establish the preferred sites of ubiquitination of NEDD4L to be in the second WW-linker (2,3-linker). Interestingly, NEDD4L ubiquitinates the cytoplasmic linker between the first and second transmembrane domains of the channel (DI-DII) of Na_V_1.5. Moreover, we design a genetically encoded modulator of Nav1.5 that achieves Na^+^ current reduction using the NEDD4L HECT domain as cargo of a Na_V_1.5-binding nanobody. These investigations elucidate the mechanisms regulating the NEDD4 family and furnish a new molecular framework for understanding Na_V_1.5 ubiquitination.

Ubiquitination is a type of posttranslational modification that is critical for maintaining cellular protein homeostasis and regulation of misfolded or mutated proteins. In the ubiquitination cascade, a target substrate is covalently tagged with a small protein called ubiquitin (Ub). The Ub modification of a substrate can happen either as a monomer (mono-ubiquitination) or as a linear/branched polymer (poly-ubiquitination) and in turn, targets substrates to the proteasome for degradation, lysosome for recycling, and/or alter protein location and function. The three main families of the ubiquitin E3 ligases are classified based on their mechanism of action. The HECT-type E3 ligases have an active site cysteine in the HECT domain and receive the Ub from the E2 enzyme, forming an E3∼Ub intermediate before transferring the Ub to a substrate protein (1). Of the 28 HECT-type E3 ligases, the NEDD4 (neuronal precursor cell-expressed developmentally down-regulated protein 4) family of enzymes have received considerable attention due to their physiological importance in regulating ion channels, stem cell differentiation, and immune activation (2, 3). The nine members of the NEDD4 family consist of an N-terminal C2 domain, 2 to 4 substrate-binding WW domains, and the catalytic HECT domain (3, 4, 5). The HECT N-lobe contains the E2 enzyme-binding site as well as the ubiquitin-exosite, which has been shown to allosterically bind Ub and support poly-ubiquitin modification (6, 7, 8, 9). The HECT C-lobe possesses the active site cysteine residue that catalyzes the Ub transfer (1, 10, 11). The two lobes are connected by a short, flexible hinge-like linker that enables a conformational switch of the HECT domain between two potential catalytically relevant conformations: an inverted T-shape and an L-shape (12, 13, 14, 15).

The regulation of HECT domain conformational change is essential for catalytic activity and substrate targeting. In this regard, NEDD4 family enzymes WWP1, WWP2, and ITCH contain a regulatory motif composed of a 30 amino acid linker between the WW2 and WW3 domains (2,3-linker) that folds as an α-helix to restrict the enzyme to its catalytically inactive T-shape conformation (16, 17, 18). In contrast, SMURF2 (19, 20) has been shown to be regulated by the C2 domain, while the NEDD4 E3 ligase is coregulated by the C2 domain and the linker region between the WW1 and WW2 domains (1,2-linker) (21). Notably, the mechanism of NEDD4L, which has the closest sequence homology to NEDD4, has not been fully understood. The NEDD4L 2,3-linker has been implicated in its regulation; however, this analysis performed exclusively in cellular experiments and lacked mechanistic biochemical investigation (22). Prior studies have also suggested that the N-terminal C2 domain regulates NEDD4L activity similarly to that described for SMURF2 (19, 23) and as a multilock mechanism where the C2, WW1 domain, and 1,2-linker pack up against the N-lobe of the HECT domain (17, 23). Despite the availability of preliminary biochemical analysis, the activation mechanism, substrate targeting, and ubiquitination site-specificity of NEDD4L are poorly understood.

In this broader context, NEDD4L has been implicated in heritable hypertensive-disease Liddle Syndrome (24, 25), hypertension, and cardiac dysfunction (26, 27). Specifically, mutations of the canonical NEDD4 family PY-binding motif of the ENaC channel leads to the disruption of NEDD4L binding and a pathogenic increase in ENaC cell surface expression that causes ionic imbalance and hypertension (24, 25). In cardiomyocytes, NEDD4L regulates the voltage-gated sodium channel Na_V_1.5, which underlies the initiation of the cardiac action potential (28). Arrhythmias in heart failure have been associated with action potential prolongation and late sodium current, though this mechanism is not fully understood (29, 30, 31, 32). Elevated NEDD4L expression and decreased Na_V_1.5 protein levels have been reported in volume-overload heart failure rat models, suggesting that NEDD4L-mediation of ubiquitination of Na_V_1.5 may contribute to the pathophysiology of heart failure (33).

The Na_V_1.5 channel is composed of a large alpha subunit that forms the ∼227 kDa ion channel pore and one or more auxiliary beta subunits. The Na_V_1.5 alpha subunit contains a ∼25 kDa extended CTerm tail that binds various cytoplasmic channel–interacting proteins which modulate the behavior of the channel (34). The Na_V_1.5 CTerm (residues 1773–2016) contains a PY NEDD4 recognition motif; PPSY residues 1974 to 1977 (35). Cell experiments and studies with patient data show that when the Na_V_1.5 PY motif is mutated, specifically Y1977, the interaction with NEDD4L is disrupted (28, 33, 36). Loss of the NEDD4L–Na_V_1.5 interaction results in elevated Na_V_1.5 protein levels and increased or leaky Na_V_1.5-mediated sodium current, which has been shown to underlie the cardiac arrhythmogenic Long QT-type 3 (LQT3) syndrome (36, 37). These examples of disease-associated mutations drive the development of molecular tools that do not rely on the PY motif.

This investigation examines the auto-regulatory mechanism of NEDD4L and its effect on targeting of the Na_v_1.5 channel substrate. We have determined the sites of ubiquitination for NEDD4L and Na_V_1.5, giving insight into how mutations alter the Ub modification or downstream recognition to disrupt normal physiology. We designed genetically encoded modulators by targeting Na_V_1.5 with nanobodies carrying the NEDD4L HECT domain as a cargo, even in the absence of the Na_V_1.5 PY motif. Our work reveals new insights into the regulation of the NEDD4 family of HECT-type E3 ligases and functional impact of ubiquitination of Na_V_1.5 channels.

## Results

### NEDD4L autoregulation is directed by the C2 domain and linker between the WW1/WW2 domains

**Figure 1:**
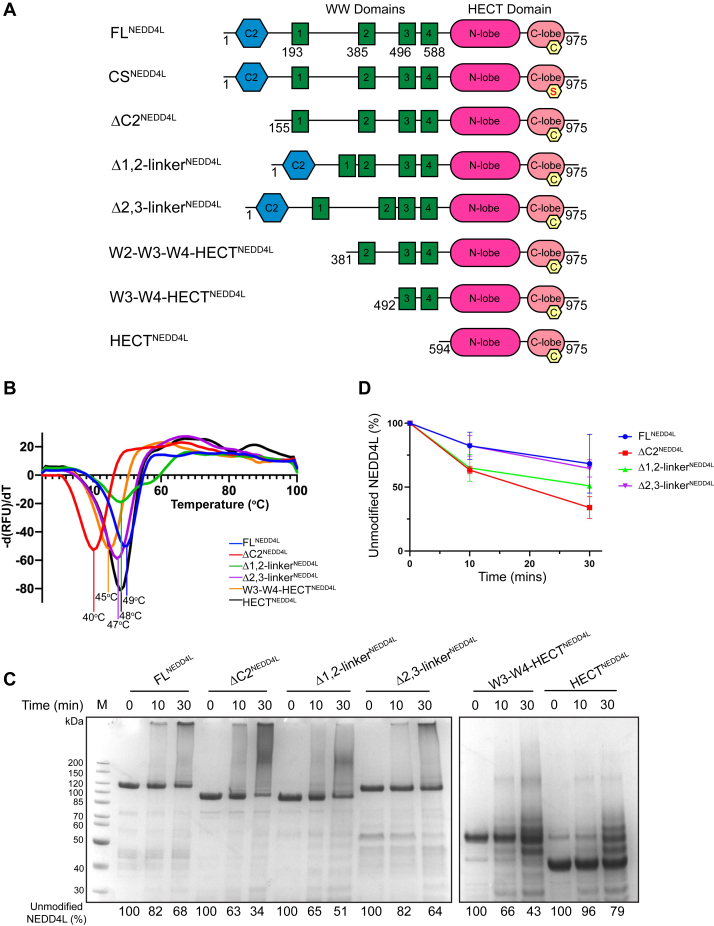
**C2 domain and 1,2-linker regulate the auto-ubiquitination of NEDD4L.***A*, schematic representation of NEDD4L variants; FL^NEDD4L^ (aa 1–975), CS^NEDD4L^ (aa 1–975, catalytically relevant residue Cys942 mutated to a Ser), ΔC2^NEDD4L^ (aa 155–356/377–975), Δ1,2-linker^NEDD4L^ (aa 1–226/383–975), Δ2,3-linker^NEDD4L^ (aa 1–418/496–975), W2-W3-W4-HECT^NEDD4L^ (aa 381–975), W3-W4-HECT^NEDD4L^ (aa 492–975), and HECT^NEDD4L^ (aa 594–975). The C2 domain is shown in *blue*; WW domains in *green*; HECT domain in shades of *pink* with the active site cysteine residue highlighted by a *yellow* pentagon. *B*, DSF thermal stability curves of the NEDD4L variants. Melting temperatures (Tm) were identified by calculating the average negative first derivative. Each Tm at the minimum is listed. DSF experiments were performed in triplicate with mean values plotted. *C*, *in vitro* ubiquitination assays of FL^NEDD4L^, ΔC2^NEDD4L^, Δ1,2-linker^NEDD4L^, Δ2,3-linker^NEDD4L^, W3-W4-HECT^NEDD4L^, and HECT^NEDD4L^. Samples were quenched with reducing loading buffer at the indicated time points. The amount of unmodified NEDD4L proteins, quantified by a densitometry analysis as a function of time, is shown as a percentage averaging all replicates. The average percentages ± SD for NEDD4L proteins are as follows (%): 100, 82 ± 10, 68 ± 23; 100, 63 ± 3, 34 ± 8; 100, 65 ± 10, 51 ± 17; 100, 82 ± 8, 64 ± 7; 100, 66 ± 7, 42 ± 9; 100, 96 ± 4, 79 ± 10. All the assays were repeated at least twice (N ≥ 2). *D*, densitometry analysis of the unmodified NEDD4L variants FL^NEDD4L^, ΔC2^NEDD4L^, Δ1,2-linker^NEDD4L^, Δ2,3-linker^NEDD4L^ from (*C*) and colored as in (*B*). Error bars represent SD. DSF, differential scanning fluorimetry.

To decode the role of each NEDD4L domain in the auto-regulatory mechanism, we designed, expressed in *Escherichia coli*, and purified six constructs with various domain deletions as well as the catalytically inactive CS^NEDD4L^ mutant (Fig. 1*A*). We deleted both linker domains between the WW1/WW2 (1,2-linker) and WW2/WW3 domains (2,3-linker) to assess their role as possible analogous enzymatic brakes observed in WWP2 and ITCH (16, 18). The thermal stability of each construct was evaluated by differential scanning fluorimetry. All NEDD4L protein variants but ΔC2^NEDD4L^ showed a melting temperature (T_m_) between 48 and 50 °C (Fig. 1*B*), close to the melting temperature of FL^NEDD4L^. Interestingly, ΔC2^NEDD4L^ showed the lowest T_m_ at 40 °C, suggesting that removal of the C2 domain disrupts the overall stability of NEDD4L (Fig. 1*B*).

Evaluation of the enzymatic activity of each variant was determined by *in vitro* auto-ubiquitination assays. We employed three techniques to measure the amount of unmodified NEDD4L protein and track the appearance of ubiquitination patterns. We carried out SDS-PAGE stained with colloidal blue as has been done previously (Fig. 1*C*) (17), analyzed ubiquitination by Western blot, and analyzed ubiquitinated bands by LC/MS/MS. Identifiers for ubiquitination can be observed by the appearance of NEDD4L bands + n∗9 kDa (where n = 1, 2, 3, etc for mono, di, and tri-Ub, respectively). Also, a smear pattern at >200 kDa are generally indication of poly-ubiquitinated NEDD4L chains. This type of ubiquitination pattern was very evident when comparing the activity of FL^NEDD4L^ to the catalytically inactive CS^NEDD4L^ mutant (Cys942Ser). *In vitro* ubiquitination assays with FL^NEDD4L^ showed a smear pattern at >200 kDa while in the presence of CS^NEDD4L^, ubiquitination was abolished, with no evidence of a smear pattern (Fig. S1*A*). Assays were performed in replicates offering varying percentages of unmodified protein levels but the trend remained the same (Fig. 1*D*).

The auto-ubiquitination assays of FL^NEDD4L^, ΔC2^NEDD4L^, Δ1,2-linker^NEDD4L^, and Δ2,3-linker^NEDD4L^ showed a smeared ubiquitin pattern >200 kDa (Fig. 1*C*) while the W3-W4-HECT ^NEDD4L^ and HECT ^NEDD4L^ proteins catalyzed smaller ubiquitin chain formations (mono, di, tri-Ub). The lack of smeared high molecular weight ubiquitination for W3-W4-HECT ^NEDD4L^ and HECT ^NEDD4L^ was interpreted as change in activity due to the absence of the regulatory domains in these constructs that lied N-terminal of the WW3 domain. Previous studies have also shown that when other NEDD4 family enzymes contained their regulatory domains, the main product was a high molecular weight smear (17, 21). The *in vitro* assay using FL^NEDD4L^ showed a steady decrease in the unmodified protein (68% at 30 min) and a high molecular weight entity that was not able to enter the SDS-PAGE (Fig. 1*C*). Deletion of the C2 domain increased NEDD4L ubiquitination activity, as previously shown (17). At 30 min, the unmodified ΔC2^NEDD4L^ was 34% less than that observed for FL^NEDD4L^ and there was evidence of a more intense ‘smear’ ubiquitination pattern. On average, the rate of which unmodified Δ1,2-linker^NEDD4L^ protein decreased was comparable to ΔC2^NEDD4L^ (Fig. 1*D*). However, we observed a different type of smear pattern when the 1,2-linker was deleted, suggesting that this region also plays a possible regulatory role (Fig. 1*C*). Interestingly, the removal of the 2,3-linker did not accelerate NEDD4L activity. In fact, activity of the Δ2,3-linker^NEDD4L^ construct seemed to be comparable to FL^NEDD4L^, given the quantification of the unmodified NEDD4L bands (64% at 30 min). Together, the autoregulated conformation involves either or both the C2 and 1,2-linker domains of NEDD4L.

To investigate whether the C2 domain and 1,2-linker worked independently or synergistically, we designed a NEDD4L construct that removed both domains and started N-terminal to the WW2 domain (W2-W3-W4-HECT^NEDD4L^, Fig. 1*A*). *In vitro* ubiquitination assays using Δ1,2-linker^NEDD4L^, ΔC2^NEDD4L^, and W2-W3-W4-HECT^NEDD4L^ were carried out for an extended time to better highlight the difference in ubiquitination patterns. The W2-W3-W4-HECT^NEDD4L^ protein experienced a rapid enhancement in the rate of ubiquitination compared to the individually domain-deleted constructs (Fig. S1*B*). By 60 min, almost all unmodified W2-W3-W4-HECT^NEDD4L^ had disappeared (Fig. S1*B*). Moreover, a greater difference in ubiquitination patterns were observed between ΔC2^NEDD4L^ and Δ1,2-linker^NEDD4L^. Bands corresponding to mono-, di-, and tri-Ub were seen with ΔC2^NEDD4L^ as early as 10 min, whereas with Δ1,2-linker^NEDD4L^, mainly mono-Ub modification was observed. In regards to high molecular weight product distributions, at 90 min, the poly-ubiquitinated forms of Δ1,2-linker^NEDDL^ entered the SDS-PAGE and ran around 200 kDa (Fig. S1*B*). On the other hand, ΔC2^NEDD4L^ poly-ubiquitinated forms mostly remain in the stacking part of the SDS-PAGE. These data argue that ΔC2^NEDD4L^ and Δ1,2-linker^NEDD4L^ have a synergistic effect, further highlighting their combined role in regulating NEDD4L.

### The C2 and 1,2-linker domains inhibit E2-E3 transthioesterification

Next, we sought to understand the effect of the C2 domain and 1,2-linker on the rate of ubiquitin transfer from the E2 to E3 enzymes which may be influenced by the conformational change of the HECT domain. For this, pulse-chase assays were performed where the E2 enzyme (UbcH5c) was charged with fluorescein-labeled ubiquitin (FAM-Ub) by incubation with E1, MgCl_2_, and ATP to form an E2(Cys)∼Ub thioester intermediate product (Fig. S2*A*). The E2(Cys)∼Ub charging reaction was quenched with EDTA to titrate out Mg^2+^, following which the indicated E3 ligases were added for the single turnover ubiquitin transfer. Assays were performed on ice with samples taken at longer time points as it was challenging to capture the E3(Cys)∼Ub intermediate with a short window of 1 min which is more commonly performed (17, 18, 38). Considering the fact that, after the E3(Cys)∼Ub to E3(Lys)-Ub transfer, the E3 can then be charged again by a new E2(Cys)∼Ub molecule in this pulse-chase experiments, we monitored the disappearance of the existing E2∼Ub to evaluate the overall E2-E3 ubiquitin transfer rate. As expected, in the absence of an E3 ligase, the natural thiolysis of E2∼Ub was very slow at the chosen time points, with only ∼20% decreasing after 15 min (Fig. S2*B*).

To determine if the transthioesterification assays could indeed differentiate and highlight a domain of regulation among NEDD4 enzymes, we performed these assays with two other NEDD4 family E3 ligases, WWP2 and NEDD4. Both enzymes were chosen because the regulatory mechanisms have been extensively assessed enzymatically (16, 21) showing the 2,3-linker autoinhibits WWP2, whereas the 1,2-linker autoinhibits NEDD4. We designed two constructs for both WWP2 and NEDD4, one of the full-length protein and the other with the auto-regulatory helical-linker deleted (Fig. S2, *C* and *D*). Removal of the 2,3-linker (Δ2,3-linker^WWP2^) enhanced the stimulation of the E2∼Ub conjugate compared to FL^WWP2^ (with 99% transfered at 5 min and 36% transfered by 10 min, respectively) (Fig. S2*E*). The unregulated Δ2,3-linker^WWP2^ protein was modified with a FAM-Ub as early as 3 min, with a more intense signal than that observed with FL^WWP2^ (Fig. S2*E*). Excess DTT was added to the reactions to confirm that the FL^WWP2^∼Ub and Δ2,3-linker^WWP2^∼Ub observed was modified on a lysine residue (Fig. S2*F*). A similar activation phenomenon of the E2∼Ub transfer was observed for the NEDD4 variants. With FL^NEDD4^, the E2∼Ub conjugate steadily decreased, taking 10 min to transfer 27% of the ubiquitin (Fig. S2*G*). Removal of the 1,2-linker decreased the time to 5 min at which ∼90% of E2∼Ub was transferred (Fig. S2*G*). Addition of DTT to the transthioesterification assays with Δ1,2-linker^NEDD4^ retained the E3-Ub band (Fig. S2*H*). These data supports the already determined auto-regulatory domains of both enzymes stimulating E2∼Ub transfer, thus supporting the use of this assay in the investigation of NEDD4L.

Four NEDD4L constructs were employed to assess the effect of the removal of the C2 domain or the two WW-linkers using transthioesterification assays as performed above. In the presence of FL^NEDD4L^, the E2∼Ub conjugate decreased with 80 to 90% transferred at 15 min (Fig. S3*A*). Removal of either the C2 domain or 1,2-linker enhanced the disappearance of the E2∼Ub conjugate, with Δ1,2-linker^NEDD4L^ being the most efficient with ∼90% transferred by 5 min (Fig. S3*A*). Deletion of the C2 domain moderately stimulated the E2∼Ub transfer compared to Δ1,2-linker^NEDD4L^. In contrast, when exposed to Δ2,3^NEDD4L^, the E2∼Ub conjugate disappeared at a rate similar to FL^NEDD4L^ (Fig. S3*A*). These results suggest that the 1,2-linker is the main contributor in the inhibition of the E2-E3 transfer, while the C2 domain appears to play an ancillary but significant role unlike the 2,3-linker. Importantly, all four NEDD4L variants were modified with a FAM-Ub as early as 5 min with ΔC2^NEDD4L^ and Δ1,2-linker^NEDD4L^ showing the most intense bands, confirming the modification on lysine residues (Fig. S3*B*).

### Ubiquitin exosite influences NEDD4L regulation

**Figure 2:**
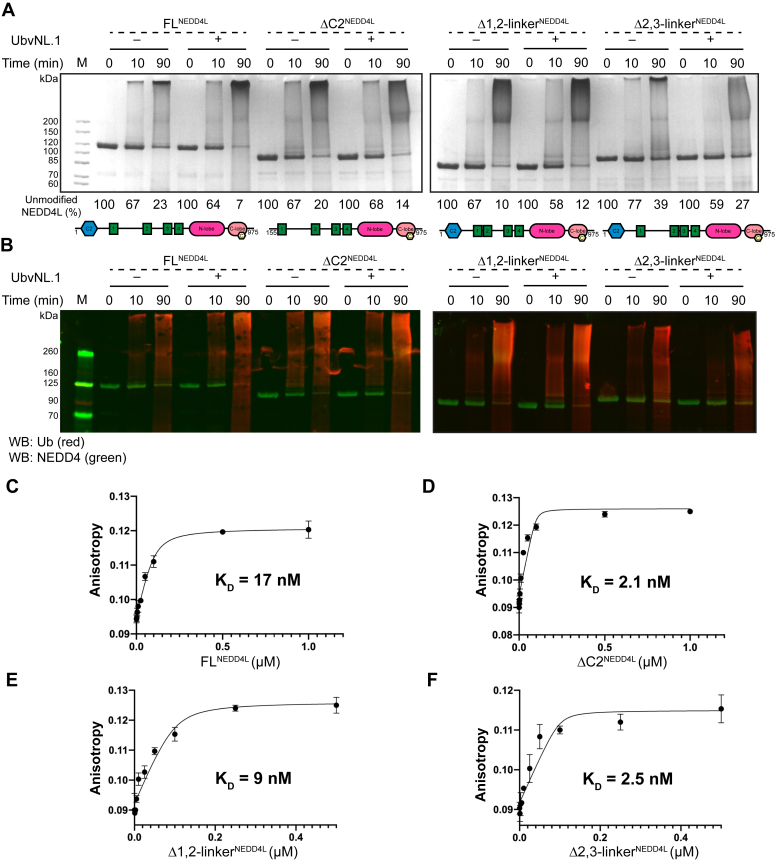
**Ubiquitin exosite in the HECT domain influences NEDD4L regulation.***A*, *in vitro* ubiquitination assays of FL^NEDD4L^, ΔC2^NEDD4L^, Δ1,2-linker^NEDD4L^, and Δ2,3-linker^NEDD4L^ in the presence and absence of 5 μM ubiquitin variant, UbvNL.1. Samples were quenched with reducing loading buffer at indicated time points. The amount of unmodified NEDD4L proteins, quantified by a densitometry analysis as a function of time, is shown as a percentage averaging all replicates. The average percentages ± SD for NEDD4L proteins in the absence and presence of UbvNL.1 are as follows (%): 100, 67 ± 1, 23 ± 4; 100, 64 ± 1, 7 ± 5; 100, 67 ± 19, 20 ± 1; 100, 68 ± 9, 14 ± 1; 100, 67 ± 7, 10 ± 7; 100, 58 ± 1, 12 ± 4; 100, 100, 77 ± 9, 39 ± 5; 100, 59 ± 21, 27 ± 14. All the assays were repeated at least twice (N ≥ 2). *B*, fluorescent Western blot analysis of the *in vitro* ubiquitination assays of FL^NEDD4L^, ΔC2^NEDD4L^, Δ1,2-linker^NEDD4L^, and Δ2,3-linker^NEDD4L^ as carried out in (*A*) using anti-ubiquitin (*red*) and anti-NEDD4 (*green*) antibodies. N = 2. *C*–*F*, binding of fluorescein-labeled UbvNL.1 to the NEDD4L variants measured by fluorescence anisotropy. Each concentration is shown with ± S.D; N = 2. The K_d_ values of UbvNL.1 for each NEDD4L variant obtained using a quadratic fit are (*C*) 17 nM with FL^NEDD4L^; (*D*) 2.1 nM with ΔC2^NEDD4L^; (*E*) 9 nM with Δ1,2-linker^NEDD4L^; (*F*) 2.5 nM with Δ2,3-linker^NEDD4L^.

To further parse the role of the NEDD4L C2 domain and 1,2-linker, we utilized fluorescence anisotropy-binding assays and Western blots in the presence of a ubiquitin variant selective for NEDD4L to better understand processivity. Zhang *et al.* (39) developed ubiquitin variants for members of the NEDD4 family of E3 ligases and showed they bind with high affinity to the HECT domain exosite and found variant UbvNL.1, modulating the activity of NEDD4L. Therefore, we sought to determine if the modulator UbvNL.1 variant could shed light on the mechanism of regulation of NEDD4L. For this, *in vitro* ubiquitination of the NEDD4L variants in the absence and presence of UbvNL.1 were analyzed by both Colloidal blue staining of SDS-PAGE and Western blot developed using fluorescent anti-Ub (red) and anti-NEDD4 (green) (Fig. 2, *A* and *B*). Importantly, in the absence of UbvNL.1, the ubiquitination patterns described in Figure 1*C* were well represented in the Western blot highlighted by the anti-Ub red signal (Fig. 2*B*). ΔC2^NEDD4L^ and Δ1,2-linker ^NEDD4L^ had a more intense smear pattern at 90 min than FL^NEDD4L^. Moreover, there was clear evidence of free Ub chains being formed as the reaction time proceeded (Source Data).

As expected, addition of UbvNL.1 activated the auto-ubiquitination of FL^NEDD4L^ (Fig. 2*A*). At 90 min, a large percentage of the unmodified FL^NEDD4L^ had been depleted (93% disappeared) (Fig. 2*A*). The rate of disappearance of the FL^NEDD4L^ band in the presence of UbvNL.1 was fast compared to when using the deletion variants in both the absence and presence of UbvNL.1 (Fig. 2*A*). The main difference observed between the NEDD4L variants in the presence of UbvNL.1 was centered around the ubiquitination distribution pattern. Specifically, in the presence of UbvNL.1, the ubiquitination pattern of FL^NEDD4L^ looked similar to that observed with ΔC2^NEDD4L^ in the absence of UbvNL.1 (Fig. 2, *A* and *B*), suggesting the C2 domain may occupy the Ub exosite. Interestingly, the ΔC2^NEDD4L^ ubiquitination product in the presence of UbvNL.1 was similar to Δ1,2-linker ^NEDD4L^ product distribution in the absence of UbvNL.1, especially the intensity of the smear pattern (Fig. 2*B*). These data strongly suggests that there could be a region other than the C2 domain partially occupying the exosite. Visual interpretation of the smear pattern of ubiquitination of Δ1,2-linker ^NEDD4L^ at 90 min in the presence or absence of UbvNL.1 showed no clear differences (Fig. 2*B*). However, at 10 min, we observed a brighter signal in the form of a smear pattern in the Western blot detection of Δ1,2-linker ^NEDD4L^ with UbvNL.1. Moreover, UbvNL.1 changed the processivity pattern of the Δ2,3-linker ^NEDD4L^ protein as well, with the ubiquitin distribution running closer to ∼200 kDa similarly to ΔC2 ^NEDD4L^ in the presence of UbvNL.1 (Fig. 2*B*).

Next, a fluorescein-labeled UbvNL.1 was produced to measure the exosite-binding affinities of FL^NEDD4L^, ΔC2^NEDD4L^, Δ1,2-linker ^NEDD4L^, and Δ2,3-linker^NEDD4L^ using fluorescence anisotropy. The affinities of FL^NEDD4L^, ΔC2^NEDD4L^, Δ1,2-linker ^NEDD4L^, and Δ2,3-linker^NEDD4L^ for UbvNL.1 were very strong and showed K_d_ values of 17, 2.1, 9, and 2.5 nM, respectively (Fig. 2, *C*–*F*). The 8-fold increase in the affinity of ΔC2^NEDD4L^ suggests that the ubiquitin exosite is obstructed when the C2 domain is present (Fig. 2*D*). The removal of the 1,2-linker only increased the binding affinity by two-fold (9 nM *versus* 17 nM), consistent with the enzymatic results where both the 1,2-linker and C2 domain influence the exosite but the C2 domain is more dominant (Fig. 2*E*). Unexpectedly, UbvNL.1 binds to Δ2,3-linker^NEDD4L^ as strongly as it does to ΔC2^NEDD4L^. Taken together, the binding properties of UbvNL.1 and the change in the ubiquitin processivity suggests the C2 domain and at least a portion of the 1,2-linker occupy the Ub exosite. Interestingly, the similarity in the high affinity and smear pattern for both the Δ2,3-linker^NEDD4L^ and ΔC2^NEDD4L^ suggests those domains behave similarly when exposed to a modulator.

### NEDD4L 2,3 linker bears the preferred auto-ubiquitination sites

**Figure 3:**
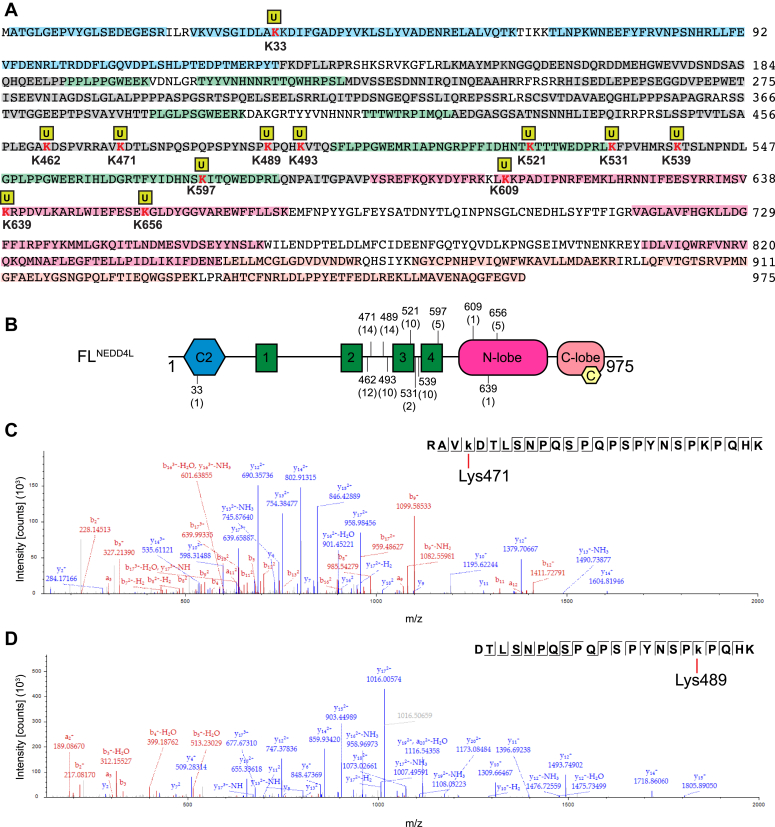
**Site-specificity of NEDD4L.***A*, FL^NEDD4L^ sequence coverage after *in vitro* band excision. Identified peptides are highlighted in *shaded regions* and colored based on the domain scheme in Figure 1*A*. *Bold*, *red letters* with a *yellow square* above them are identified with Lys sites with a Gly-Gly modification. *B*, domain scheme of FL^NEDD4L^ with the Lys sites of ubiquitination labeled based on location. The peptide spectrum matches of each Lys residue is in parentheses. *C*, representative MS/MS spectrum and sequence coverage of the peptide containing a Gly-Gly modification on Lys471 and (*D*) Lys489. Lower case k indicates a Gly-Gly modification.

To further assist in the understanding of the auto-regulation mechanism, we sought to determine by mass spectrometry the FL^NEDD4L^ lysine sites of ubiquitination as well as the ubiquitin chain linkage assembled. For this, an *in vitro* reaction using FL^NEDD4L^ was carried out for 60 min and the high molecular weight ‘smear’ observed in the stacking part of the SDS-PAGE (between the comb and ∼200 KDa) was excised. In-gel enzymatic digestion was performed using trypsin followed by LC/MS/MS analysis. Since the amino group of a lysine residue forms an isopeptide bond with the C-terminus glycine residue of ubiquitin, a Gly-Gly (GG) modification will be observed in the MS analysis if a lysine residue has a ubiquitin molecule attached. Of the 48 lysines within FL^NEDD4L^, the majority are located within the C2 and HECT domains, with no lysine residues present in the 1,2-linker. MS analysis revealed an 86% sequence coverage of FL^NEDD4L^ and identified 12 lysine residues with a Gly-Gly modification (Fig. 3*A*). The identified sites were mainly localized between residues 460 and 670 which encompasses the 2,3-linker, WW3, and WW4 domains as well as a portion of the N-lobe of the HECT domain (Fig. 3*B*). The lysine residues with the highest spectral counts were observed in the 2,3-linker, making up approximately 60% of the total number detected (Fig. 3*B*). Moreover, Gly-Gly modifications were detected on each lysine residue (Lys462, Lys471, Lys489, and Lys493) in the 2,3-linker (Fig. 3, *C* and *D*).

The MS analysis assessing the ubiquitin linkage assembled by FL^NEDD4L^ revealed a Gly-Gly modification on five of the seven lysine residues of ubiquitin (Lys6^Ub^, Lys11^Ub^, Lys33^Ub^, Lys48^Ub^, Lys63^Ub^) (Fig. S4*A*). The modified Ub lysine residues with the highest spectral counts were Lys63 and Lys48, constituting approximately 63% and 25%, respectively (Fig. S4, *B* and *C*). To further parse the two types of ubiquitin chains, we performed *in vitro* assays with mutant ubiquitin, specifically Lys48^Ub^ and Lys63^Ub^ mutated to an arginine residue (K48R^Ub^, K63R^Ub^) (Fig. S4*D*). No change in the ubiquitination chain pattern or amount of unmodified FL^NEDD4L^ protein was observed using K48R^Ub^ compared to WT ubiquitin (Fig. S4, *D* and *E*). In contrast, a rapid enhancement in the ubiquitination of FL^NEDD4L^ was observed when using K63R^Ub^ (Fig. S4, *D* and *E*). Interestingly, we observed the appearance of shorter Ub chains (mono-, di-, and tri-Ub) as early as 10 min with K63R^Ub^, suggesting that Lys48 makes short Ub modifications in the absence of the preferred Lys63. While this result was not unexpected as many NEDD4 family members catalyze Lys63 linkages, establishment of auto-ubiquitination chains can provide insight into the chains formed on essential substrates (40). Taken together, the 2,3-linker of NEDD4L is essential for robust ubiquitination, and the assembly of Lys48^Ub^ and Lys63^Ub^ linkages at varying chain lengths gives insight into possible targeting of the E3 ligase.

### NEDD4L catalytic activity downregulates Na_V_1.5 protein levels

**Figure 4:**
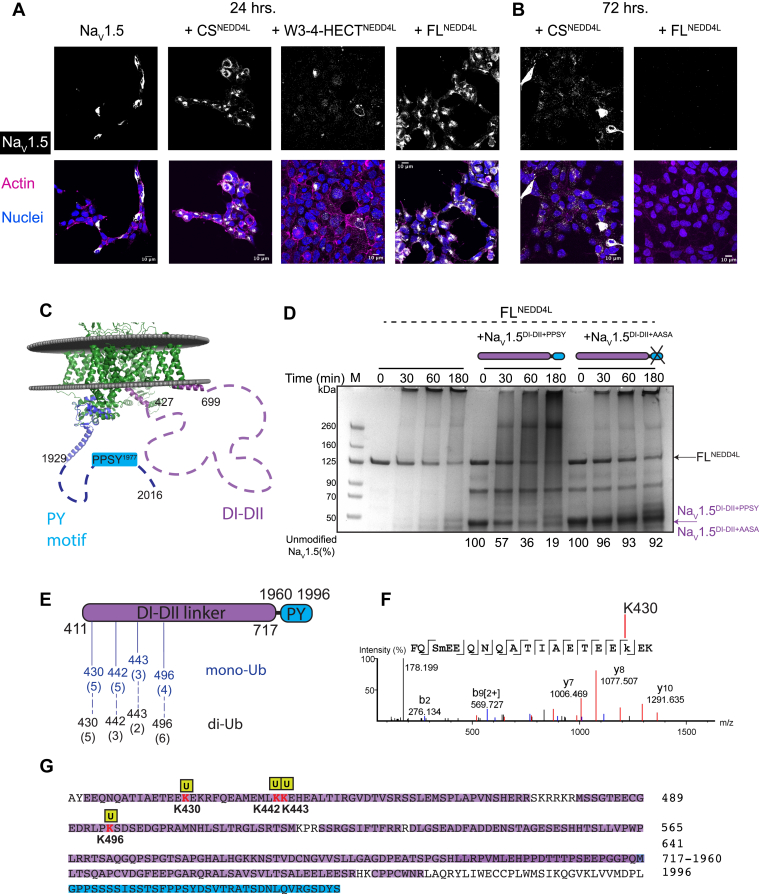
**Na**_**V**_**1.5 is ubiquitinated by FL**^**NEDD4L**^**on Na**_**V**_**1.5 DI-DII linker.***A*, HEK293 cells transfected with Na_V_1.5 alone or cotransfected with CS^NEDD4L^, W3-4HECT^NEDD4L^, or FL^NEDD4L^ and cultured for 24 or (*B*) 72 h. Cells were stained with anti-Na_V_1.5 antibody (*white*), phalloidin dye to mark actin filaments (*magenta*), and Hoescht3342 to mark nuclei (*blue*). *C*, Na_V_1.5 chimeric construct spans DI-DII linker (*purple*; residues 411–717) and CTerm extended PY motif (*blue*; residues 1960–1996). Composite of the existing cryo-EM structure of Na_V_Pas (Na_V_ of American cockroach *Periplaneta americana*; PDB ID 5X0M; *green*) aligned with experimental structure of Na_V_1.5^CTerm^ (PDB ID 4OVN; *blue*). *Dashed lines* indicate regions lacking published structural data (each dash ∼10 amino acids). *D*, time course of the *in vitro* ubiquitination of FL^NEDD4L^ in the absence and presence of Na_V_1.5^DI-DII linker+PPSY^ or Na_V_1.5^DI-DII linker+AASA^ substrate. Equal amounts of samples were taken at the indicated time points and quenched with reducing loading buffer. The SDS-PAGE was stained with colloidal Coomassie blue stain. The amount of unmodified Na_V_1.5^DI-DII linker^, quantified by a densitometry analysis as a function of time, is shown as a percentage averaging all replicates. The average ± SD for Na_V_1.5^DI-DII linker+PPSY/AASA^ protein in the presence of NEDD4L enzyme are as follows (%): 100, 57 ± 6, 36 ± 6, 19 ± 5, 100, 96 ± 4, 93 ± 7, 92 ± 9. All the assays were repeated at least twice (N ≥ 2). *E*, scheme of Na_V_1.5^DI-DII+PPSY^ with the Lys sites of ubiquitination labeled based on location. Mono- and di-Ub Na_V_1.5^DI-DII+PPSY^ bands were excised from 3 h *in vitro* gel lane. The LC/MS/MS peptide spectrum matches of each Lys residue are in *parentheses*. *F*, representative MS/MS spectrum and sequence coverage of the peptide containing a Gly-Gly modification on Na_V_1.5^DI-DII+PPSY^ Lys430. Lower case k indicates a Gly-Gly modification and lower case m indicates oxidized methionine. *G*, Na_V_1.5^DI-DII+PPSY^ sequence coverage after band excision from the *in vitro* assay. Identified peptides are highlighted in *shaded regions* and colored based on the domain scheme in (*D*).

To fully understand the role that individual NEDD4L domains play in the auto-inhibition mechanism, we assessed their spatial and temporal effects on the ubiquitination of the substrate Na_V_1.5. Although reports show that the pore-forming alpha subunit of Na_V_1.5 is regulated by NEDD4L and that their interaction is mediated by the PPSY motif located on the Na_V_1.5 cytoplasmic CTerm (28, 33, 35) (Fig. S5*A*), biochemically the mechanism of regulation, sites of ubiquitination, and chain linkage have yet to be determined. To visualize NEDD4L regulation of Na_V_1.5 in a cellular context, we overexpressed Na_V_1.5 in the absence and presence of FL^NEDD4L^ and the active form, W3-W4-HECT^NEDD4L^ in HEK293 cells. As ubiquitin modification most often results in targeting of the substrate for recycling, we performed immunofluorescent staining of Na_V_1.5 and detected the remaining protein levels by confocal microscopy. Imaging of the cells transfected with Na_V_1.5 alone after 24 h revealed evident levels of Na_V_1.5 (Fig. 4*A*). Cotransfection with FL^NEDD4L^ did not result in a noticeable difference in protein levels after 24 h (Fig. 4*A*) but rendered Na_V_1.5 almost undetectable by 72 h (Fig. 4*B*). Interestingly, after just 24 h, the Na_V_1.5 signal was almost completely depleted when cotransfected with the active W3-W4-HECT^NEDD4L^ construct (Fig. 4*A*). However, cotransfection with catalytically inactive CS^NEDD4L^, where the active site cysteine residue is mutated to a serine (Fig. 1*A*), exhibited a much attenuated decrease in Na_V_1.5 (Fig. 4, *A* and *B*). These results confirm that the levels of Na_V_1.5 at the membrane are regulated by the catalytic activity of NEDD4L.

### Na_V_1.5 is ubiquitinated on its DI-DII linker

To identify the Na_V_1.5 sites of ubiquitination, we ran UbPred software (41) using the sequence of human Na_V_1.5 (Uniprot: Q15858), which contains a total of 83 lysine residues. Out of these, 13 are located in the intracellular linker between the DI and DII transmembrane domains (Na_V_1.5^DI-DII^), eight of which were predicted to be ubiquitinated with medium to high confidence. Notably, no lysine residue in the Na_v_1.5 CTerm was predicted to be ubiquitinated. Despite this indication, since the CTerm contains the canonical PPSY NEDD4L recognition motif, we assessed its possible *in vitro* ubiquitination by NEDD4L (Figs. 4*C* and S5, *B* and *C*). Substrate ubiquitination reactions were carried out under the same conditions as the auto-ubiquitination *in vitro* reactions, solely with the addition of the substrate (up to 3 h at 30 °C) and in parallel with FL^NEDD4L^ auto-ubiquitination reactions for comparison. The FL^NEDD4L^ (Fig. S5*B*, lanes 2–5) displayed a steady decrease of unmodified protein from 30 min to 3 h, an increase of high molecular weight poly-ubiquitinated species, and significant free Ub chains in the 25 to 50 kDa range. At the same time, addition of the Nav1.5^CTerm^ substrate did not result in the decrease of unmodified Nav1.5^CTerm^ nor the appearance of Nav1.5^CTerm^ + n∗9 kDa Ub bands as expected if ubiquitination occurred (Fig. S5, *B* and *C*). Rather, we only observed a decrease of unmodified FL^NEDD4L^ as well as free Ub chains comparable to the reaction in the absence of substrate.

To determine which residues were indeed Ub-modified, we selected the Na_V_1.5^DI-DII^ spanning residues 411 to 717 for further *in vitro* evaluation as suggested by the *in silico* prediction. To promote FL^NEDD4L^ recognition of Na_V_1.5^DI-DII^ *in vitro*, we hypothesized that the PPSY motif located in the Na_V_1.5^CTerm^ was required. Therefore, we designed a chimera with the Na_V_1.5^DI-DII^ (residues 411–717) fused to the extended PPSY motif (residues 1960–1996), termed Na_V_1.5^DI-DII+PPSY^. Moreover, to confirm the role of the PPSY motif in FL^NEDD4L^ recognition, we mutated the PPSY motif to AASA in the chimera. We performed *in vitro* ubiquitination assays with FL^NEDD4L^ in the absence and presence of substrate Na_V_1.5^DI-DII+PPSY^ and Na_V_1.5^DI-DII+AASA^ (Fig. 4*D*). The *in vitro* assay, in the absence of Na_V_1.5^DI-DII+PPSY^, displayed the typical auto-ubiquitination pattern. In the presence of Na_V_1.5^DI-DII+PPSY^, it showed the appearance of an intense, poly-ubiquitinated smear pattern above the Na_V_1.5^DI-DII+PPSY^ chimeric protein (Fig. 4*D*), particularly at 3 h (Fig. 4*D*). A steady decrease in the unmodified Na_V_1.5^DI-DII+PPSY^ was also observed over 3 h (100% at 0 min, 57% at 30 min, and 19% after 3 h) (Fig. 4*D*). Side-by-side enzymatic assays of Na_V_1.5^DI-DII+PPSY^ and Na_V_1.5^DI-DII+AASA^ highlighted the absence of ubiquitination patterns (Fig. 4*D*), especially the poly-Ub smear in the presence of the alanine-mutated Na_V_1.5^DI-DII+AASA^. The single band observed above the unmodified Na_V_1.5^DI-DII+AASA^ at 3 h was also present in the auto-ubiquitination control of FL^NEDD4L^ without Na_V_1.5, indicating the band represents free ubiquitin chains (Fig. 4*D*, lane 13). Moreover, the unmodified Na_V_1.5^DI-DII+AASA^ protein decreased only to 96% at the 30 min time point compared to the 57% of Na_V_1.5^DI-DII+PPSY^ protein, confirming the importance of the PPSY motif for NEDD4L recognition.

### Na_V_1.5 DI-DII-linker N-terminal preferred sites of ubiquitination displays Lys63 linkage

With the observation of strong ubiquitination of the Na_V_1.5^DI-DII+PPSY^ protein, we determined the preferred lysine sites as well as ubiquitin chain linkage by mass spectrometry. In order to generate intense, individual bands corresponding to Na_V_1.5^DI-DII+PPSY^ +9 kDa and +18 kDa for band excision, an *in vitro* assay was performed in 3× excess of Na_V_1.5^DI-DII+PPSY^ (Fig. S5, *D* and *E*). The mono- and di-Ub bands were excised from the 3 h SDS-PAGE gel lane and confirmed by mass spectrometry to be the mono- and di-Ub-modified Na_V_1.5^DI-DII+PPSY^, respectively. The MS analysis indicated ubiquitin-modification on Na_V_1.5^DI-DII+PPSY^ N-terminal residues Lys430, Lys442, Lys443, and Lys496 for both mono- and di-Ub-Na_V_1.5^DI-DII+PPSY^ (Figs. 4, *E* and *F* and S5, *F*–*H*). Moreover, the sequence coverage of the Na_V_1.5^DI-DII+PPSY^ chimera was 95 to 96% for each ubiquitinated band (Fig. 4*G*). Furthermore, all seven lysine residues of ubiquitin were detected by MS analysis and ubiquitin (Gly-Gly) modification was identified on lysine residues Lys48^Ub^ and Lys63^Ub^ (Fig. S6*A*). Residue Lys63^Ub^ yielded the majority of the observed Gly-Gly modifications, ∼87% of the spectral count (Fig. S6, *B*–*D*).

To further investigate both ubiquitin linkages assembled on the Na_V_1.5^DI-DII+PPSY^ chimera, we performed the *in vitro* ubiquitination of FL^NEDD4L^ and Na_V_1.5^DI-DII+PPSY^ substrate and analyzed the pattern by Western blot with antibodies specific for Lys63- (Fig. S6, *E* and *F*) and Lys48-linked Ub (Fig. S6, *G* and *H*). The FL^NEDD4L^ auto-ubiquitination reaction (Fig. S6, *E*–*H*, lanes 2–5) showed steady increase of high molecular weight signal when stained with the Lys63^Ub^ antibody (Fig. S6*E*). In contrast, the Lys48-linked Ub blot showed distinct lower molecular weight (40–70 kDa) bands from 1 to 3 h corresponding to free Ub chains (Fig. S6*G*), further suggesting that the shorter Ub modifications are through Lys48^Ub^ linkage as was shown with the Ub mutants. Na_V_1.5^DI-DII+PPSY^ appears to be prominently poly-ubiquitinated by Lys63^Ub^ chains by the appearance of a bright smear pattern between its unmodified molecular weight and ∼260 kDa (Fig. S6*E*, lanes 6–9), supporting the LC/MS/MS analysis. Comparison of the blots suggests more abundant Lys63^Ub^ *versus* Lys48^Ub^ linkages in the substrate Na_V_1.5^DI-DII+PPSY^ modifications in congruence with the number of spectral counts observed. The total protein stains (Fig. S6, *F* and *H*) show clear bands for Ub, free diUb, E2 enzyme, Na_V_1.5^DI-DII+PPSY^, and FL^NEDD4L^ in the assay similar to the Colloidal blue–stained SDS-PAGE.

### NEDD4L intramolecular interactions dictate Na_V_1.5 ubiquitination

We further probed the functional role of the NEDD4L regulatory domains in substrate ubiquitination both biochemically and cellularly. First we ran *in vitro* assays with Na_V_1.5^DI-DII+PPSY^ in the presence of the FL^NEDD4L^, ΔC2^NEDD4L^, Δ1,2-linker ^NEDD4L^, and Δ2,3-linker ^NEDD4L^ variants (Fig. S7*A*). Ubiquitin modifications of Na_V_1.5^DI-DII+PPSY^ such as mono- and di-Ub bands were observed at similar levels for all four NEDD4L variants (Fig. S7*A*). Western blot analyses of these *in vitro* assays displayed a similar higher MW ubiquitinated smear pattern for both where the NEDD4L variants and Na_V_1.5^DI-DII+PPSY^ proteins were located (Fig. S7*B*).

**Figure 5:**
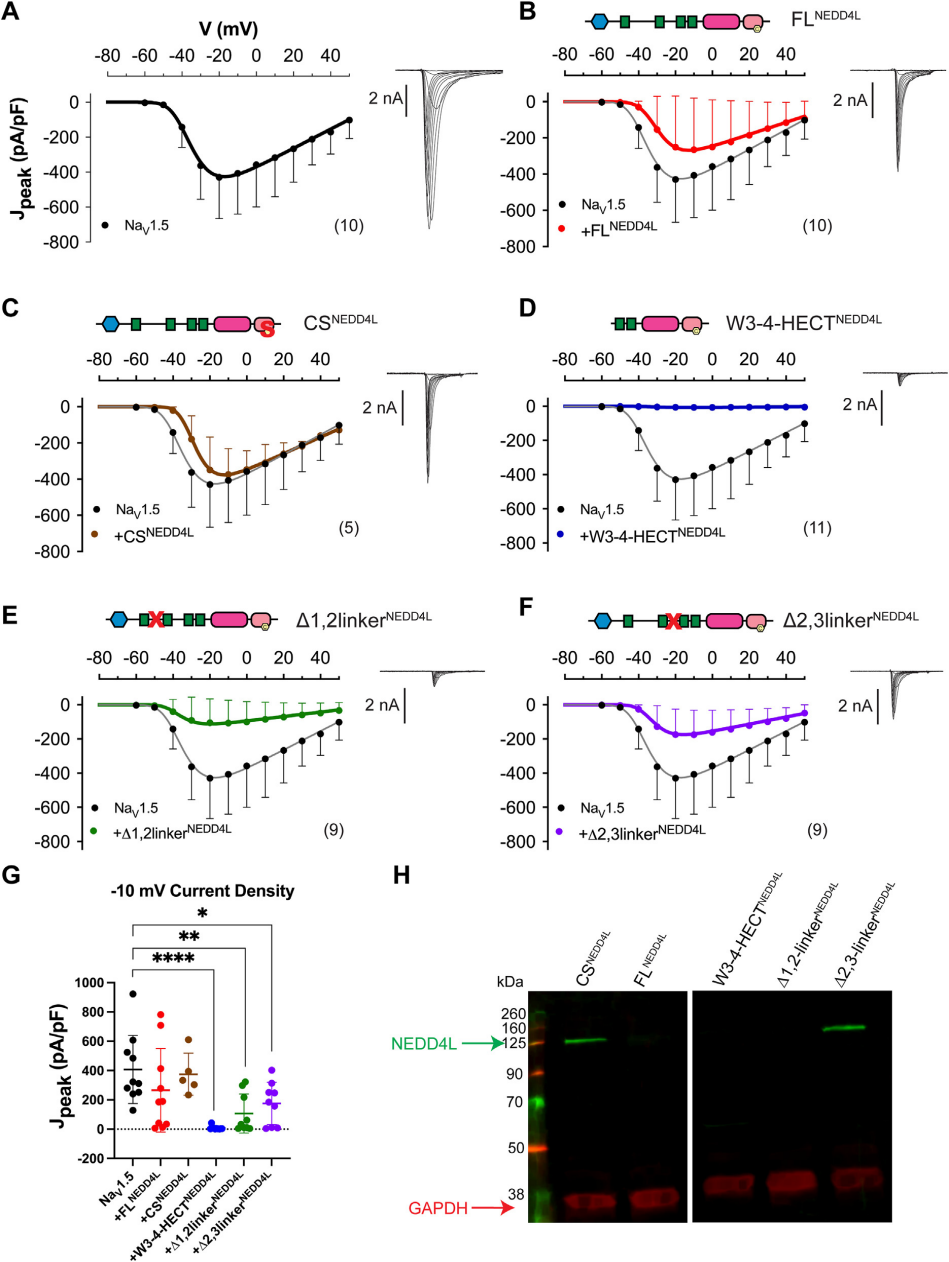
**Cotransfection with NEDD4L variants reduces Na**_**V**_**1.5-mediated sodium current.***A*, average peak current density (*J*_peak_) – voltage relationship from wild-type Na_V_1.5 channels elicited in response to a family of 10 ms steps from −60 to +50 mV from a holding potential of −120 mV. Each dot, mean ± SD with n denoted in *parenthesis*. *B*–*F*, J_peak_ for Na_V_1.5 (*black*) and upon cotransfection with NEDD4L variants (*B*) FL^NEDD4L^, (*C*) CS^NEDD4L^, (*D*) W3-4-HECT^Nedd4L^, (*E*) Δ1,2-linker^NEDD4L^, (*F*) Δ2,3-linker^NEDD4L^, (*G*) *J*_peak_ at −10 mV. Mean ± SD, ∗*p* < 0.05, ∗∗*p* < 0.01, ∗∗∗∗*p* < 0.0001 by one-way ANOVA with Tukey’s multiple comparisons test. *H*, representative Western blot showing immunodetection of the NEDD4L variant protein levels (*green*) used in (*B*–*F*) and GAPDH (*red*; loading control) in HEK293. NEDD4L antibody epitope is the HECT domain present in all NEDD4L variants. Four such experiments yielded comparable results.

With that, we then sought to probe the functional consequences of NEDD4L autoregulation on Na_V_1.5 currents. Using the NEDD4L variants FL^NEDD4L^, CS^NEDD4L^, Δ1,2-linker ^NEDD4L^, Δ2,3-linker^NEDD4L^, and W3-W4-HECT^NEDD4L^, we performed whole-cell patch-clamp recordings to evaluate Na_V_1.5-mediated sodium current (I_Na_) in HEK293 cells. At baseline, transfection of Na_V_1.5 alone showed peak current density (*J*_peak_) of −407 ± 73 pA/pF (mean ± s.e.m, n = 10) at −10 mV membrane potential (Fig. 5, *A* and *G*). Cotransfection with FL^NEDD4L^ yielded a minor ∼35% reduction in the peak sodium current density that was not statistically significant (Fig. 5, *B* and *G*). Further quantification of the voltage-dependence of activation revealed minimal changes upon co-expression of FL^NEDD4L^ (Fig. S7*C*). Co-expression of catalytically inactive CS^NEDD4L^ also revealed minimal changes in I_Na_ (Fig. 5, *C* and *G*). By comparison, cotransfection with W3-W4-HECT^NEDD4L^, which lacks all the auto-inhibitory motifs, virtually eliminated the sodium current (*p* < 0.0001 relative to Na_V_1.5; Fig. 5, *D* and *G*). In like manner, cotransfection with Δ1,2-linker^NEDD4L^, which showed accelerated ubiquitination *in vitro* compared to FL^NEDD4L^, strongly decreased peak current density by ∼75% (*p* = 0.0039 relative to Na_V_1.5; Fig. 5, *E* and *G*). The Δ2,3-linker^NEDD4L^ also reduced the peak sodium current density by ∼56% (*p* = 0.0356 relative to Na_V_1.5; Fig. 5, *F* and *G*).

Notably, when we performed Western blot analysis to compare the decrease in sodium current with levels of NEDD4L protein, only CS^NEDD4L^ and Δ2,3-linker^NEDD4L^ were detectable 24 h after transfection of HEK293 (Fig. 5*H*). This is congruent with the NEDD4L auto-regulatory mechanism; CS^NEDD4L^ lacks the enzymatic ability to transfer Ub, and the Δ2,3-linker^NEDD4L^ lacks the preferred sites of Ub modification (Fig. 2*A*); thus both variants would be deficient in auto-ubiquitination and not be degraded (3, 42). The enduring CS^NEDD4L^ and Δ2,3-linker^NEDD4L^ levels over 24 h would result in increased Na_V_1.5 substrate modification corresponding to decreased I_Na_ for Δ2,3-linker^NEDD4L^ but not the inactive CS^NEDD4L^. Taken together with the *in vitro* results, the NEDD4L 1,2-linker is central in regulation of the substrate and auto-modification activity.

### NanoMaNs modulate Na_V_1.5-mediated Na^+^ current

**Figure 6:**
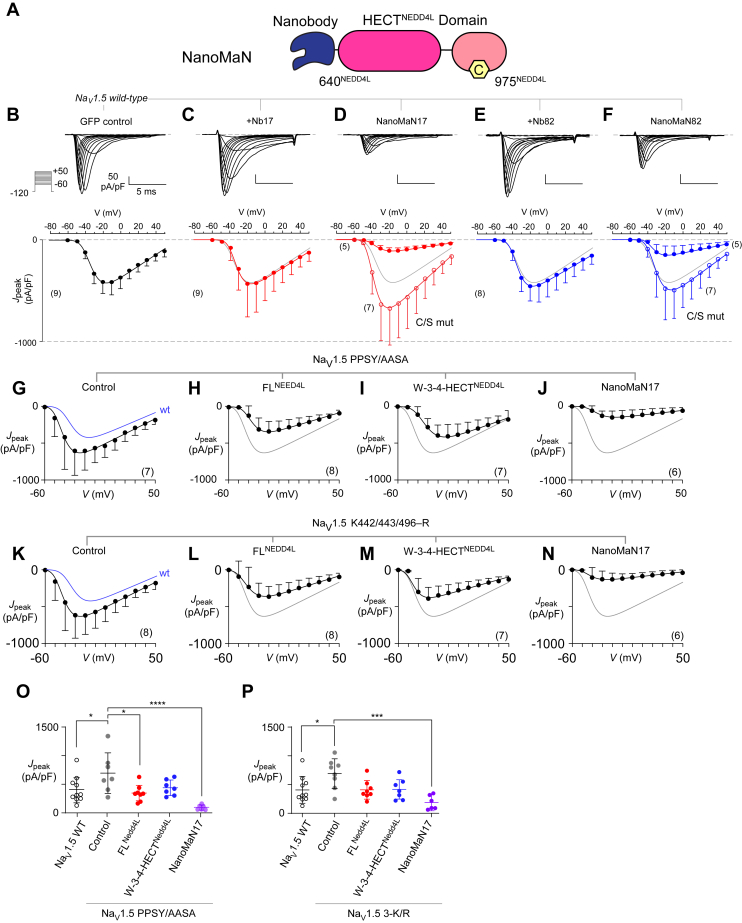
**NanoMaN targets Na**_**V**_**1.5 to diminish I**_**Na**_**.***A*, schematic of the NanoMaN construct composed of an N-terminal nanobody (either Nb17 or Nb82) and the catalytic HECT domain of NEDD4L comprising residues 640 to 975. *B*, *top*, exemplar current recordings for WT Na_V_1.5 channels elicited in response to a family of voltage steps to −60 to +50 mV from a holding potential of −120 mV. *Bottom*, population data shows average peak current density (*J*_peak_) – voltage relationship. Each dot, mean ± SD with n denoted in *parenthesis*. *C*, co-expression of Nb17 alone yielded minimal change in peak Na current density. *D*, overexpression of both NanoMaN17 diminished peak current densities. Format as in panel (*B*). *E*, Nb82 alone also minimally perturbed peak current density. *F*, NanoMaN82 also diminished peak current density. *G*, average peak current density (*J*_peak_) – voltage relationship for Na_V_1.5 PPSY/AASA mutant channels. Each dot, mean ± SD with n denoted in parenthesis. *H*–*J*, *J*_peak_ for Na_V_1.5 PPSY/AASA mutant channels upon co-expression of NEDD4L variants (*H*) FL^NEDD4L^, (*I*) W3-4-HECT^Nedd4L^, and (*J*) NanoMan17. *K*, average peak current density (*J*_peak_) – voltage relationship for Na_V_1.5 K442/443/496–R mutant channels. Each dot, mean ± SD with n denoted in *parenthesis*. *L*–*N*, *J*_peak_ for NaV1.5 PPSY/AASA mutant channels upon co-expression of NEDD4L variants (*L*) FL^NEDD4L^, (*M*) W3-4-HECT^NEDD4L^, and (*N*) NanoMan17. *O*, bar graph summarizes changes in *J*_peak_ for Na_V_1.5 PPSY/AASA mutant in the presence of NEDD4L variants. Mean ± SD, ∗*p* < 0.05, ∗∗*p* < 0.01, ∗∗∗∗*p* < 0.0001 by one-way ANOVA with Dunnett’s multiple comparisons test. *P*, bar graph summarizes changes in *J*_peak_ for Na_V_1.5 K442/443/496–R mutant in the presence of NEDD4L variants. Mean ± SD, ∗*p* < 0.05, ∗∗∗*p* < 0.001 by one-way ANOVA with Dunnett’s multiple comparisons test. NanoMaN, nanobody modulators of Na+ channels.

With a better understanding of the importance of the NEDD4L-Na_V_1.5 PY motif for ubiquitination, we sought to investigate an alternative way of recognition in its absence, for example, when disease-associated PY mutations are present (28, 33). Since nanobodies have been shown to be molecular tools that bind with high affinity to a target antigen and are an effective way to deliver cargo (43), we chose our selected nanobodies Nb17 and Nb82 that specifically recognize Na_V_1.5 (44). We designed two chimeric constructs which fused the coding sequence Nb17 (or Nb82) to a cargo, the NEDD4L catalytic HECT domain. We term these probes nanobody modulators of Na^+^ channels (NanoMaNs). We hypothesized that upon transfection, NanoMaNs would bind the Na_V_1.5^CTerm^ independent of the PY-motif, bringing the HECT domain in proximity to ubiquitinate the channel complex (Fig. 6*A*). To test this hypothesis, we performed whole-cell patch-clamp electrophysiology in HEK293 cells co-expressing Na_V_1.5 with NanoMaNs or nanobodies alone and recorded peak current densities (Fig. 6). We observed robust Na_V_1.5 currents upon expression of GFP as a negative control (*J*_peak_ = −416 ± 42 pA/pF; mean ± s.e.m, n = 9; Fig. 6*B*). Co-expression of Nb17 or Nb82 alone yielded no appreciable change in peak current densities (Fig. 6, *C* and *E*). However, co-expression of NanoMaN17 evoked a marked ∼75% reduction in peak current density (*p* = 0.02 compared to Nb17 alone; Fig. 6*D*). Similarly, NanoMaN82 yielded a ∼70% reduction in peak current density (*p* = 0.03 compared to Nb82 alone; Fig. 6*F*).

Thus assured, we sought to determine whether the NanoMaN approach regulated channels independently of the the canonical PY-motif and if so, whether this process required ubiquitination of the DI-DII linker. To do so, we substituted the Na_V_1.5^CTerm^ PPSY-motif with AASA residues yielding full-length Na_V_1.5 PPSY/AASA. This maneuver has been shown to disrupt the Na_V_–NEDD4L interaction and regulation of Na_V_1.5 current density (35). We observed a basal increase in *J*_peak_ of the Na_V_1.5 PPSY/AASA mutant channel compared to WT Na_V_1.5 (Fig. 6, *G* and *O*). Furthermore, co-expression of FL^NEDD4L^ and the hyperactive W-3-4-HECT^NEDD4L^ mutant yielded a mild reduction or no change in *J*_peak_ (Fig. 6, *H*, *I* and *O*), suggesting that the Na_V_1.5^CTerm^ PPSY-motif is critical for the recruitment of NEDD4L to the channel complex. Importantly, NanoMaN17 strongly diminished *J*_peak_ (85% reduction; Fig. 6, *J* and *O*), suggesting that targeted recruitment of the NEDD4L catalytic domain to the channel complex suffices for functional regulation. Thus informed, we probed whether the NanoMaN approach also relied on ubiquitination of the DI-DII linker. To do so, we mutation substituted Lys442, Lys443, and Lys496 with Arg and measured changes in *J*_peak_. Like the PPSY/AASA mutant channel, the NaV1.5 K442/443/496-R mutant also exhibited basal increase in *J*_peak_ (Fig. 6, *K* and *P*). Co-expression of FL^NEDD4L^ and hyperactive W-3-4-HECT^NEDD4L^ yielded mild but not statistically significant changes in *J*_peak_ (Fig. 6, *L*, *M* and *P*), confirming that these residues in the DI-DII linker are indeed critical for functional channel regulation by NEDD4L. Furthermore, co-expression of NanoMan17 reduced *J*_peak_ by ∼72%. Of note, in all cases, we once again observed minimal changes in voltage-dependence of activation (Fig. S7, *C*–*E*).

These finding suggests that ubiquitination of the DI-DII linker is not necessary when NEDD4L is recruited to the channel complex using the nanobody. Instead, NanoMaN17 may co-opt alternate Lys residues to downregulate channel function. One possibility is that the Na_V_1.5^CTerm^ PPSY motif and the DI-DII linker may be spatially apposed to facilitate ubiquitination of Lys442, Lys443, and Lys496. As Nanobody17 interacts *via* a distinct interface, other Lys residues elsewhere on the channel may become accessible for ubiquitination enabling channel regulation.

## Discussion

**Figure 7:**
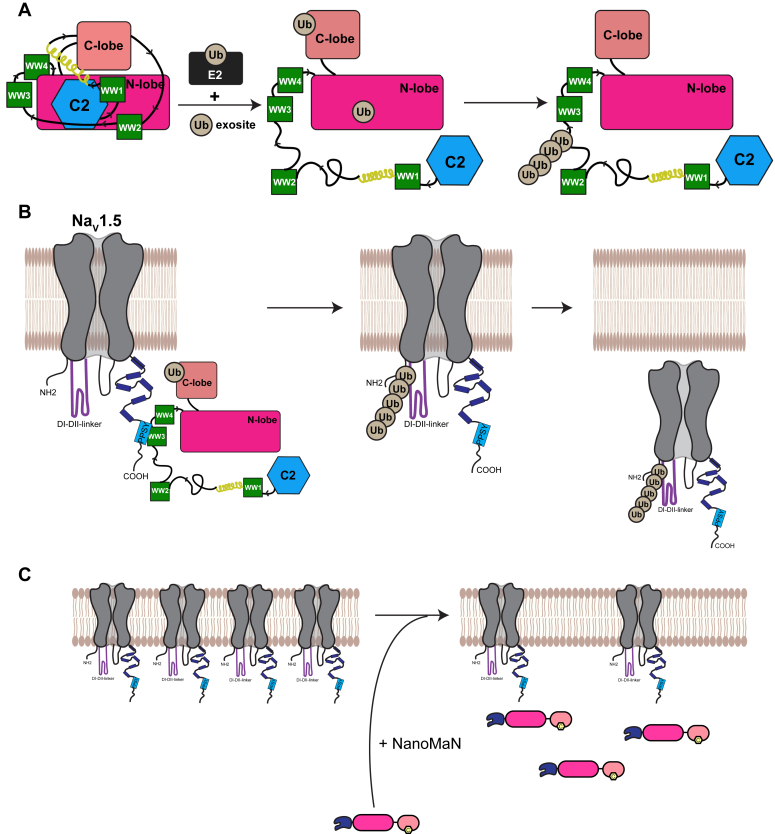
**Proposed NEDD4L and Na**_**V**_**1.5 mechanism of regulation.***A*, the intramolecular interactions of the NEDD4L C2 domain and 1,2-linker lock the HECT domain in the inactive T-shape. Upon ubiquitin exosite binding, the regulatory domains are released and the HECT domain transitions into the active L-shape. Subsequent auto-ubiquitination can occur on the preferred lysine residues within the 2,3-linker. *B*, the NEDD4L recognition motif located in the CTerm of the voltage-gated sodium channel Na_V_1.5 binds to the WW3 or WW4 domains. Substrate binding near the 1,2-linker can initiate regulation release and robust ubiquitination. Specifically, for Na_V_1.5, the ubiquitination occurs in the DI-DII-linker; not the region that has the recognition motif. *C*, NanoMans reduced the peak sodium current of Na_V_1.5 suggesting less Na_V_1.5 channels at the plasma membrane. NanoMaN, nanobody modulators of Na+ channels.

In this study, we show that NEDD4L, a HECT-type E3 ligase, is auto-regulated by both the C2 domain and the linker between the WW1 and WW2 domains (1,2-linker), with the latter playing a more significant role in the voltage-gated sodium channel Na_V_1.5 substrate targeting (Fig. 7). While this was not an unanticipated mechanism based on previous studies, our data further highlights many new principles. First, the auto-regulation of NEDD4L *via* the C2 domain and 1,2-linker involves the occupancy of the ubiquitin exosite in the HECT domain and further highlights how the 2,3-linker could be important for placement of the C2 domain (Fig. 7*A*). Second, the preferred sites of ubiquitination lie within the linker between the WW2 and WW3 domains (2,3-linker) highlighting its overall importance to NEDD4L E3 ligase activity (Fig. 7*A*). Third, NEDD4L ubiquitinates Na_V_1.5 in the DI-DII cytoplasmic linker and not in the Na_V_1.5^CTerm^, which contains the NEDD4 recognition motif (Fig. 7*B*). This ubiquitination is regulated primarily by the 1,2-linker of NEDD4L with Lys63 as the preferred residue for Na_V_1.5-ubiquitin chain linkage. Fourth, NanoMaNs have the potential to be used as molecular tools to target Nav1.5 upon disease-associated PY motif mutations (Fig. 7*C*).

Of note, an important caveat is that our electrophysiological measurements quantified peak current density of Na_V_1.5. Mechanistically, this change in peak current density could stem from altered channel trafficking to the surface membrane or from a change in the intrinsic channel activity. Eventhough, the 35% reduction in current in the presence of FL^NEDD4L^ is much smaller than observed with the NEDD4L mutant forms in which autoinhibition is relieved, we attempted to quantify surface expression. Those attempts following Tarradas *et al.* (45), Bio-protocol 2013 were unsuccessful. Specifically, we biotinylated Na_V_1.5 channels in HEK293 cells, performed subsequent pull-down experiments using neutravidin ultralink resin, and probed for yellow fluorescent protein (YFP) expression of the YFP tagged Na_V_1.5 channel. Although we were able to observe the YFP signal from a positive control, we encountered challenges in detecting YFP-Na_V_1.5 signals. We speculate the lack of detection might be due to the low abundance of surface expression of Na_V_1.5. Further studies quantifying surface membrane expression of Na_V_1.5 and complementary single channel analysis would be necessary to unambiguously establish the mechanism underlying peak current density changes.

In the past couple of years, the auto-regulatory mechanism of NEDD4L has been investigated. Barring structural data, many have used pull-down and *in vitro* ubiquitination assays in the presence of binding partners and modulators of E3 ligase activity to better understand where the intramolecular interactions that rule regulation are located. Mari *et al.* (20) characterized the role of the C2 domain on Smurf2 and NEDD4-1 through NMR analysis as well as highlighted that the C2 domain has an overlapping binding interface to that of the noncovalent ubiquitin exosite. Our *in vitro* ubiquitination and binding assays further suggest that the mechanism is similar for NEDD4L with both the C2 domain and 1,2-linker lying near the ubiquitin exosite which is located on the ‘front’ of the HECT domain N-lobe (Fig. 7*A*). Since the binding affinity of UbvNL.1 to the HECT domain is higher in the absence of the C2 domain compared to the 1,2-linker, the C2 domain likely occupies majority of the exosite, while the 1,2-linker occupies less exosite surface. While the C2 domain binds in the ‘front’ of the NEDD4L HECT domain, Wang *et al.* (17) suggested, through pull-down assays, that the WW1 domain binds the ‘back’ side of the HECT domain and a portion of the 1,2-linker ‘wraps’ around the HECT hinge region. We show that removal of the WWP2 2,3-linker and NEDD4 1,2-linker substantially increased the rate of ubiquitin transfer, highlighting free rotational change of the HECT domain. Since the N-terminal of the NEDD4L 1,2-linker is predicted to be helical, similar to the 2,3-linker of WWP2 and ITCH, it is possible that a similar interaction occurs. The activated transfer rate with Δ1,2-linker^NEDD4L^ supports that this region is an analogous enzymatic brake, wrapping around the HECT domain. While the C2 domain also shows optimal transfer, it is possible that the interaction of the C2 domain with the Ub exosite anchors the 1,2-linker in place and its removal disrupts 1,2-linker–hinge interaction. Moreover, the reduction of Na_V_1.5-mediated inward sodium current was statistically significant when Δ1,2^NEDD4L^ was co-expressed relative to FL^NEDD4L^. Our data suggests that the 1,2-linker is a possible anchoring point for the HECT domain and the Na_V_1.5 PPSY motif has been shown to interact with the WW3^NEDD4L^ and WW4^NEDD4L^ domains (46). If the WW domains are in proximity to the 1,2-linker^NEDD4L^, engaging the Na_V_1.5 PPSY motif may release intramolecular interactions of NEDD4L. This in turn would release auto-inhibition and activate substrate targeting (Fig. 7).

Until now, the sites of ubiquitination of NEDD4L were not known and the role of the NEDD4L 2,3-linker was not as well characterized. The *in vitro* assays consistently showed a slower rate of ubiquitination in the presence of the Δ2,3-linker^NEDD4L^ variant, suggesting inhibition of NEDD4L activity upon removal. The mass spectrometry analysis revealed the 2,3-linker contains the preferred sites of Ub modification, highlighting that upon the 2,3-linker deletion, the transfer of ubiquitin could be slower due to ubiquitination of suboptimal lysine sites (Fig. 7*B*). Even though the sites were removed in the assays studying the exosite, if the 2,3-linker was not playing a role in the auto-regulation, there would be no difference in the binding and Ub pattern in the presence or absence of a modulator like UbvNL.1. However, this is not what was observed. UbvNL.1 bound Δ2,3^NEDD4L^ as tightly as ΔC2^NEDD4L^ and the ubiquitin product distributions in the presence of the UbNL.1 variant were similar. One possible explanation is that the 2,3-linker interacts with the C2 domain in a way that it anchors it into the HECT domain N-lobe. Removal of the 2,3-linker, with the addition of a competitive binding partner, could completely release the interaction of the C2 domain to the N-lobe. Interestingly, Grimsey *et al.* (22) showed that a tyrosine residue (Y485) on the NEDD4L 2,3-linker gets phosphorylated and they hypothesized that the residue interacts with an acidic triad in the closed conformation of the C-lobe of the HECT domain. With our data, it is possible that residue Y485 could be important for binding to the C2 domain and its phosphorylation causes similar activation to that observed in the presence of the ubiquitin variant.

For many years, it has been known that Na_V_1.5 is a substrate of NEDD4L (28, 35) but the biochemical characterization and Ub site identification were lacking. To date, there have been no assays illustrating *in vitro* ubiquitination of Na_V_1.5. Despite the canonical NEDD4 PPSY-binding motif being located on the CTerm cytoplasmic portion of the channel and the presence of nine lysine residues, no ubiquitination occurs *in vitro*. Instead, the Na_V_1.5 DI-DII linker chimera shows robust ubiquitination (Fig. 7*B*). The four ubiquitinated lysine residues are located downstream of the first domain, which spatially could be in close proximity to the Na_V_1.5^CTerm^ PPSY motif (Fig. 4*C*). One of the highly identified lysine residues, Lys430, is directly proximal to the Na_V_1.5 429delE mutation associated with Long QT-type 3 (37). It is possible that the deletion disrupts the secondary structure of that region, in turn affecting the Na_V_1.5-Lys430-Ub surface presented, leading to less overall ubiquitination and more Na_V_1.5 at the cell surface. Moreover, the DI-DII linker displays the greatest sequence divergence amongst the nine human Na_V_ isoforms with pairwise sequence identity between 40 and 55%, underscoring the specificity of this Ub regulation (44). Lys442, another highly ubiquitinated identified lysine residue in our study, has also been reported to be SUMOylated (47). Furthermore, the modification was both sufficient and necessary to generate a hypoxia-induced Na_V_1.5 late sodium current (I_LATE_) (47). Other reports indicate that there may be SUMO and Ub regulatory crosstalk in the context of protein degradation (48).

The importance of NEDD4L substrate recognition *via* the Na_V_1.5 PY motif is highlighted by our *in vitro* data, previous cell studies (28, 33), and the clinical prevalence of diseases associated with a disrupted PY motif. To overcome this limitation, we designed NanoMaNs as a potential modulator for targeting disease-associated Na_V_1.5 PY-mutant channels. These NanoMaNs combine the specificity of the Na_V_1.5 nanobodies with an active ‘cargo’ to facilitate channel internalization. The reduction in peak current density of Na_V_1.5 in the presence of NanoMaN17 and 82 supports the functionality of these constructs. While further work is required to determine whether the NanoMaN approach could lead to therapeutics, this study serves as a proof of principle that such an approach has the potential to mitigate the deleterious effects of channelopathic Na_V_1.5 mutations as well as aberrant sodium current in acquired heart disease.

## Experimental procedures

### Plasmids and reagents

Human ubiquitin-activating enzyme UBE1, human ubiquitin-conjugating enzyme UbcH5c, and the constructs for Ub^K48R^, Ub^K63R^ were a gift from Dr Cynthia Wolberger at Johns Hopkins University. The E1 and E2 enzyme were purified as described before (49).

### Protein expression and purification of GST-tagged NEDD4L variants

The pcDNA3.1(+) plasmid with the full-length NEDD4L human sequence (aa 1–975; Q96PU5) was purchased from Addgene and all variants of NEDD4L were subcloned by Genescript. DNA sequences coding for FL^NEDD4L^ (aa 1–975), ΔC2^NEDD4L^ (aa 155–356/377–975), Δ1,2-linker^NEDD4L^ (aa 1–226/383–975), Δ2,3-linker^NEDD4L^ (aa 1–418/496–975), and HECT^NEDD4L^ (aa 594–975) proteins were subcloned into either pGEX6p-1 or pGEX6p-2 expression vectors and transformed into BL21-CodonPlus RIL *E. coli* cells. The transformed cells were cultured in LB medium, supplemented with 100 μg/ml carbenicillin and 25 μg/ml chloramphenicol, at 37 °C to reach the optimal density (OD_600_ = 0.8) on an 8-l scale. Protein production was induced with 0.5 mM IPTG and grown overnight at 18 °C for 20 h. Cells were harvested at 4000*g* and the cell pellets were frozen at −80 °C.

Upon thawing, the cells were resuspended in lysis buffer (25 mM Hepes pH 7.5, 300 mM NaCl, 1 mM DTT) supplemented with 0.5% Triton X-100, 1 mM phenylmethylsulfonyl fluoride (PMSF), and 1× Roche cocktail protease inhibitors. Cells were lysed using a microfluidizer (Microfluidics Corporation; model 110 Y) and the lysates were clarified at 11,000 × rpm for 1 h. The supernatants were loaded onto 3 ml of GSH-agarose (pre-equilibrated with lysis buffer) using a gravity flow column, followed by washing with 25 mM Hepes pH 7.5, 300 mM NaCl, 1 mM DTT, and 0.1% Triton X-100. The desired GST-tagged proteins were eluted using 25 mM Hepes, 300 mM NaCl, 1 mM DTT, and 20 mM reduced glutathione at pH 8.0. The eluted fractions were combined and dialyzed against a buffer consisting of 25 mM Hepes pH 7.5, 50 mM NaCl, 1 mM TCEP, and the proteins were treated with PreScission protease at 4 °C overnight to cleave the GST tag. Afterward, the mixture of GST and cleaved proteins were loaded onto a Source Q anion exchange column (Cytiva). Elution was performed using the buffer 25 mM Hepes, pH 7.5 and 1 mM TCEP and a step gradient of 50 to 500 mM NaCl. Fractions were analyzed by SDS-PAGE and assessed for 98% purity. For *in vitro* and binding assays, the purified NEDD4L variants were concentrated to ∼2 to 4 mg/ml, flash frozen, and stored at −80 °C.

### Protein expression and purification of His_6_-tagged NEDD4L variants

DNA sequences coding for W2-W3-W4-HECT^NEDD4L^ (aa 381–975) and W3-W4-HECT^NEDD4L^ (aa 492–975) proteins were subcloned into pET-28a(+) expression vector and were transformed into LOBSTR *E. coli* cells. The transformed cells were cultured in LB medium, supplemented with 50 μg/ml kanamycin, at 37 °C to reach the optimal density (OD_600_ = 0.8) on an 8-l scale. Protein production was induced with 0.5 mM IPTG and grown overnight at 18 °C for 20 h. The cells were harvested, resuspended in lysis buffer (25 mM Hepes pH 7.5, 300 mM NaCl, 10 mM β-mercaptoethanol (BME)), with additives of 0.5% Triton X-100, 1 mM PMSF, 1× Roche cocktail protease inhibitors, and lysed using a microfluidizer. The cell lysates were loaded onto 2 ml of pre-equilibrated Ni-NTA agarose using a gravity flow column. The beads with bound protein were washed with 30 ml of 25 mM Hepes pH 7.5, 300 mM NaCl, 10 mM BME, and 20 mM imidazole and then 5 ml of buffer with 50 mM imidazole. The desired His_6_-tagged proteins were eluted using 25 mM Hepes pH 7.5, 300 mM NaCl, 10 mM BME, and 300 mM imidazole. The eluted fractions were analyzed by SDS-PAGE and the fractions containing the pure proteins were pooled and dialyzed against a buffer containing low salt (25 mM Hepes pH 7.5, 50 mM NaCl, 1 mM TCEP) at 4 °C overnight. For further purification, the His_6_-tagged proteins were loaded onto a Source Q anion exchange column (Cytiva). Elution of the protein was performed using the buffer 25 mM Hepes, pH 7.5 and 1 mM TCEP and a step gradient of 50 to 500 mM NaCl. Fractions were analyzed by SDS-PAGE for 98% purity. For *in vitro* and binding assays, the purified proteins were concentrated to ∼2 to 4 mg/ml, flash frozen, and stored at −80 °C.

### Protein expression and purification of WWP2 and NEDD4 constructs

pGEX6p-2 expression plasmids subcloned with either FL^WWP2^ (aa 1–870), Δ2,3-linker^WWP2^ (aa 1–361/394–870), FL^NEDD4^ (aa 1–900), or Δ1,2-linker^NEDD4^ (aa 1–224/245–900) were transformed into BL21(DE3) Codon Plus *E.coli* competent cells for recombinant protein expression. The transformed cells were cultured from fresh LB-agar plates into LB medium to optimal cell density at OD_600_ = 0.6. Then 0.5 mM IPTG was added to induce protein expression at 16 °C for 20 h. The cells were centrifuged at 5000 × rpm for 10 min, and the pellet was resuspended in a lysis buffer that contained 25 mM Hepes pH 7.8, 250 mM NaCl, 1 mM TCEP, 1 mM PMSF, and 1× Pierce EDTA-free cocktail protease inhibitor (Thermo Fisher Scientific, A32965). After resuspension, cells were lysed by french press, centrifuged, and the supernatant was loaded onto a glutathione-agarose column for binding. The resultant resin-bound mixture was then washed with 25 mM Hepes pH 7.8, 250 mM NaCl, 1 mM TCEP, and 0.1% Triton X-100. The GST-tagged protein was eluted with 12 ml of a elution buffer containing 25 mM Hepes pH 7.8, 250 mM NaCl, 1 mM TCEP, and 50 mM reduced glutathione (GoldBio, G15525). The eluted fractions were then treated with Prescission protease (Cytiva, 27084301) for the removal of the GST-tag overnight in a dialysis cassette against a buffer of 25 mM Hepes pH 7.8, 250 mM NaCl, and 1 mM TCEP. The cleavage mixture was re-applied to a glutathione agarose column to remove the cleaved GST tag and the Prescission protease. The protein was further purified with a Superdex 200 Increase 10/300 GL size exclusion column (Cytiva). The size-exclusion chromatography fractions were analyzed by Coomassie blue–stained SDS-PAGE. The corresponding fractions were combined, and glycerol was added to a final concentration of 10% v/v. The purified proteins were concentrated with Amicon, flash-frozen, and stored at −80 °C.

### WT and lysine-mutant ubiquitin protein expression and purification

DNA sequences coding for human WT and lysine-mutant (K48R, K63R) ubiquitin proteins were subcloned into pET3a and transformed into BL21(DE)3 *E. coli* cells. The transformed cells were cultured in 2-l of LB medium at 37 °C until an OD_600_ = 0.6 and protein production was initiated by the addition of 0.5 mM IPTG at 16 °C for 16 h. Upon thawing, the cells were resuspended in 50 mM Tris pH 7.6, 10 mM MgCl_2_, 0.01% Triton X-100, and 1 mM PMSF and lysed with a microfluidizer. The ubiquitin supernatants were collected, placed on ice, and precipitated by dropwise addition of 70% v/v solution of perchloric acid while stirring. Precipitation was stopped once reaching pH 4 to 5 and centrifugation was performed at 11,000 × rpm to separate out precipitate. The clarified supernatant was dialyzed overnight at 4 °C against 50 mM ammonium sulfate at pH 4.5, loaded onto a Source S cation exchange column (Cytiva), and elution was performed using a linear gradient with 500 mM NaCl in 50 mM ammonium sulfate pH 4.5. Fractions were analyzed by SDS-PAGE for 98% purity and dialyzed into 20 mM Hepes pH 7.5, 50 mM NaCl, and 0.5 mM DTT. For ubiquitination assays, purified WT and lysine-mutant ubiquitin proteins was concentrated to ∼2 to 4 mg/ml.

### Protein expression and purification of ubiquitin variant UbvNL.1

The DNA sequence for UbvNL.1 was subcloned into a pGEX6p-2 plasmid vector and then transformed into BL-21 Codon Plus RIL *E. coli* cells. The *E. coli* cells were cultured in 2-l of LB medium at 37 °C and protein production was initiated with 0.5 mM IPTG and grown overnight at 16 °C for 20 h. Collected cells were resuspended in lysis buffer (25 mM Hepes pH 7.5, 250 mM NaCl, 1 mM DTT) supplemented with 1× cocktail protease inhibitor (Thermo Fisher Scientific) and 1 mM PMSF. Cells were lysed using a microfluidizer and the lysate was clarified at 11,000 × rpm for 1 h. The supernatant was loaded onto 3 ml of pre-equilibrated GSH-agarose using a gravity flow column, followed by washing with 25 mM Hepes pH 7.5, 250 mM NaCl, 1 mM DTT, and 0.1% Triton X-100. The desired GST-tagged ubiquitin variant UbvNL.1 was eluted using 25 mM Hepes, 300 mM NaCl, 1 mM DTT, and 50 mM reduced glutathione at pH 8.0. The eluted fractions were combined and dialyzed against a buffer consisting of 25 mM Hepes pH 7.5, 250 mM NaCl, 1 mM DTT, and the protein was treated with PreScission protease at 4 °C overnight to cleave the GST tag. Afterward, size-exclusion chromatography with a Superdex 75 Increase 10/300 GL column (Cytiva) was used to further purify the protein in the running buffer 25 mM Hepes pH 7.5, 250 mM NaCl, 1 mM DTT. Purified fractions (purity >90%) were combined, concentrated, and stored at −80 °C.

### Protein expression and purification of Na_V_1.5^DI-DII+PPSY^ and Na_V_1.5^DI-DII+AASA^ proteins

The DNA sequence that codes for Na_V_1.5^DI-DII linker^ including aa 411 to 717 and the PY motif aa 1960 to 1996 were subcloned into pNIC28 expression vector downstream of a 6× His tag by GenScript; the construct was called Na_V_1.5^DI-DII+PPSY^. The plasmid Na_V_1.5^DI-DII+PPSY^ was mutated 1974 to 1977 to code for AASA for the Na_V_1.5^DI-DII+AASA^ chimera protein. BL21 *E. coli* cells were transformed with the pNIC28 plasmids expressing Na_V_1.5^DI-DII+PPSY^ and Na_V_1.5^DI-DII+AASA^ chimeras. The transformed cells were cultured in LB medium, supplemented with 50 μg/ml kanamycin, at 37 °C to reach the optimal density (OD_600_ = 0.8) on an 8-l scale. Protein production was induced with 0.5 mM IPTG and grown at 18 °C for 20 h. The cells were harvested, resuspended in lysis buffer (25 mM Tris-HCl pH 7.5, 250 mM NaCl, 10 mM BME), with additives of 1% Triton X-100, 1 mM PMSF, 10 mM imidazole, 1× mixture of protease inhibitors, and lysed using a microfluidizer. The cell lysates were loaded onto 2 ml of pre-equilibrated Ni-NTA agarose beads (Qiagen) using a gravity flow column. The beads with bound protein were washed with 50 ml of 25 mM Tris–HCl pH 7.5, 250 mM NaCl, 10 mM BME, and 10 mM imidazole. The desired His_6_-tagged proteins were eluted using 25 mM Tris–HCl pH 7.5, 250 mM NaCl, 10 mM BME, and 150 mM imidazole and then 25 mM Tris–HCl pH 7.5, 250 mM NaCl, 10 mM BME, and 500 mM imidazole. The eluted fractions were analyzed by SDS-PAGE, and the fractions containing the pure proteins were pooled and dialyzed against a buffer containing low salt (25 mM Tris–HCl pH 7.5, 50 mM NaCl, 1 mM DTT) at 4 °C overnight. For further purification, the His_6_-tagged proteins were loaded onto a Source Q anion exchange column (GE, Millipore Sigma). Elution was performed using the buffer 25 mM Tris–HCl, pH 7.5 and 1 mM DTT and a step gradient of 50 to 500 mM NaCl. Fractions were analyzed by SDS-PAGE for 80 to 90% purity. For *in vitro* and binding assays, the purified proteins were concentrated to ∼1 to 4 mg/ml, flash frozen, and stored at −80 °C.

### Protein expression and purification of the C-terminal Na_V_1.5 in complex with Calmodulin

The C-terminal domain of Na_V_1.5 (CTNa_V_1.5; aa 1775–2016) with an N-terminal GST tag was co-expressed with Calmodulin (CaM) in *E. coli* BL21(DE3) cells and purified using GST-sepharose, similar to the protocol described by Gabelli *et al.* (50) 2014 for CTNa_V_1.5-CaM (aa 1773–1929). The protein was eluted with reduced L-glutathione and the GST-tag was cleavaged with Precision Protease at 4 °C overnight. Pooled fractions were further purified by anion exchange chromatography, judged to be >95% pure by SDS-PAGE analysis, and concentrated to ∼5 mg/ml, flash frozen, and stored at −80 °C.

### *In vitro* ubiquitination assays

*In vitro* ubiquitination assays were performed in a 1 ml microcentrifuge tube at a total volume of 30 μl containing 50 nM E1, 3 μM E2 (UbcH5c), 50 μM ubiquitin (Ub), 2.5 μM NEDD4L variant proteins with 40 mM Tris–HCl, pH 7.5, 50 mM NaCl, 0.5 mM TCEP, 5 mM ATP, and 5 mM MgCl_2_. The reactions were initiated by the addition of E1 and carried out at 30 °C. The reactions were quenched at the indicated time points with the addition of 2× SDS-PAGE reducing loading buffer. The reaction samples were then boiled for 5 min at 97 °C and loaded onto a 12% SDS-PAGE gel along with a molecular weight marker (Thermo Fisher Scientific, PageRuler Unstained Protein Ladder, 3 μl). The gels were analyzed using colloidal Coomassie Blue staining following the manufacturers protocol. Briefly, the SDS-PAGE gel was first primed for staining by washing with a 50% ethanol/10% acetic acid solution for 10 min, followed by washing with distilled water for 5 min. After priming, 100 ml of a staining solution containing 10% ammonium sulfate, 2% phosphoric acid 85%, 5% CBB G-250, and 20% ethanol was added to the SDS-PAGE and incubated overnight. After sufficient destaining, the unmodified E3 or substrate protein bands for each replicate were quantified using ImageJ densitometry, normalized to the zero time point, and averaged.

### E2-E3 transthioesterification assays

The E2 conjugating enzyme, UbcH5c (15 μM), was charged with fluorescein-labeled-Ub (25 μM, FAM-Ub (51) in 40 mM TRIS pH 7.5, 50 mM NaCl, 5 mM MgCl_2_, 5 mM ATP, and 50 μg/ml bovine serum albumin (BSA)). The E2∼Ub charging reaction (10 μl) was initiated by the addition of the E1 enzyme (250 nM) and incubated at room temperature for 40 min. The charged E2 reaction was quenched with a three-fold dilution in 25 mM Hepes pH 7.5, 100 mM NaCl, 30 mM EDTA. Second, the quenched E2∼Ub was mixed with 1 μM of each indicated NEDD4L variant and 50 μM WT Ub on ice to initiate the transfer of Ub to the E3 ligase. Reactions were quenched at the indicated time points with nonreducing 2× SDS-PAGE loading buffer. The SDS-PAGE gels were analyzed by fluorescent imaging (Typhoon) and colloidal Coomassie Blue staining to visually confirm the same amount of NEDD4L variants were added to the reactions. Thioester-linked intermediates were confirmed by adding 200 mM DTT to each time point. The E2∼Ub intermediate products from each replicate were quantified using ImageJ densitometry, normalized to the zero time point, and averaged.

For the E2-E3 transthioesterificaiton assays with WWP2 and NEDD4, the E2∼Ub conjugate was made similarly to that described above. Firstly, 0.25 μM E1, 15 μM E2 (UbcH5b), and 25 μM N-terminal fluorescein-labeled ubiquitin were incubated at 25 °C for 40 min in a ubiquitination buffer containing 40 mM Tris *pH* 7.5, 50 mM NaCl, 5 mM MgCl_2_, 5 mM ATP, and 50 μg/ml BSA. After 40 min, the reaction was quenched by a five-dilution with quenching buffer that contains 25 mM EDTA, 40 mM Tris *pH* 7.5, 50 mM NaCl. Secondly, E2∼Ub was mixed with different forms of WWP2 (WT or Δ2,3-linker, 30 °C) or NEDD4 (WT or Δ1,2-linker, on ice) and WT ubiquitin (for allosteric activation) to initiate the single turnover E2 to E3 ubiquitin transfer, resulting in a mixture of 1 μM E3, 0.6 μM E2∼Ub, 10 nM E1, 1.7 μM fluorescent ubiquitin, and 400 μM wt ubiquitin. Reactions were quenched with a nonreducing NuPAGE LDS sample loading buffer (Invitrogen) at the indicated time points. The confirmation of thioester linkage was done by adding 50 μM DTT to the sample for reduction. Then, ubiquitinated products were analyzed by SDS-PAGE, and the detection of fluorescent ubiquitin was performed using a Typhoon FLA 9500 imager (GE) using the preset FAM fluorescent program.

### Generation of fluorescein-labeled ubiquitin variant F-UbvNL.1 and FAM-Ub

For UbvNL.1 labeling, Ser-57 was mutated to Cys by QuikChange mutagenesis. The ubiquitin variant mutant was expressed and purified as described above. The purified UbvNL.1 S57C protein (1 mg) was mixed with 10-fold molar excess of 5-iodoacetamidofluorescein (Thermo Fisher Scientific) in PBS buffer containing 5 mM EDTA at room temperature in the dark for 4 h. After 4 h, the excess labeling reagent was removed by dialysis and the fluorescein-labeled UbvNL.1 was further purified with Superdex 75 10/300 GL column size-exclusion chromatography using a buffer containing 25 mM Hepes, pH 7.3, 150 mM NaCl, 1 mM EDTA, 2 mM DTT, and 5% glycerol.

N-terminal FAM-labeled ubiquitin was generated as described previously (51, 52). Briefly, 6xHis-TEV-Cys-ubiquitin was expressed in Rosetta DE3 pLysS *E. coli* cells and purified using Ni^+^ NTA column. After the purification, TEV protease was added to cleave the His-tag and expose the N-terminal Cysteine for labeling. After cleavage, the proteins were re-applied to Ni^+^ NTA resin to remove 6x-His-TEV and TEV protease. Then the protein was dialyzed into 100 mM Hepes pH 7.0, 150 mM NaCl, and 1 mM TCEP for 16 h at 4 °C followed by N-terminal labeling with freshly prepared FAM fluorescein thioester for 24 h at room temperature. After the labeling reaction, the protein was dialyzed into 25 mM Hepes pH 8.0, 250 mM NaCl, and 1 mM TCEP for 16 h at 4 °C to remove most of the fluorescein thioester that remained. Next, the labeled ubiquitin was further purified by size-exclusion chromatography (Superdex 75 10/300 GL column, flowrate 0.5 ml/min) using a mobile phase of 25 mM Hepes pH 8.0, 250 mM NaCl, 10% glycerol, and 1 mM TCEP to remove residual fluorescein thioester or protein aggregates. Purified fractions (>90%, Coomassie-stained SDS-PAGE) were combined, concentrated, and stored at −80 °C until further biochemical analysis.

### Fluorescence anisotropy binding assays

F-UbvNL.1 (1 nM) was mixed with the indicated concentrations of NEDD4L variants in buffer (25 mM Tris–HCl pH 7.5, 150 mM NaCl, 1 mM EDTA, 5 mM DTT) and incubated at room temperature for 30 min in a black, flat bottom 96-well plate (Corning). Steady-state fluorescence anisotropy data were acquired using a Synergy H1 microplate reader (BioTek) at 25 °C. The excitation wavelength was set to 495 nm and the emission was measured at 520 nm. Each data points were performed in triplicate. The binding curves and K_d_ values were generated using the quadratic-binding fit with the equation Y = Y_0_ − [(Y_0_ − Y_max_)/(2∗Fixed)] ∗ [b − sqrt(b^∧^2 − 4∗X∗Fixed)] (b = K_d_ + X + Fixed, Fixed = 0.1).

### Tandem mass spectrometry

Ubiquitination sites of FL^NEDD4L^ were identified by tandem mass spectrometry (MS/MS). Samples were separated *via* SDS–PAGE and visualized with colloidal Coomassie blue staining. FL^NEDD4L^ and FL^NEDD4L^-Ub bands were excised, cut into 1 mm × 1 mm pieces, and dehydrated with methanol for 5 min. All samples were reduced with DTT, alkylated, and digested with trypsin (Promega, 12.5 ng/μl in 40 μL of 50 mM TEAB at 37 °C overnight (53)). Tryptic peptides were extracted with 50% acetonitrile, 0.1%TFA, dried, and reconstituted with 150 μl 0.1% TFA in water, acidified, and desalted on u-HLB Oasis plates (Waters). Fifteen percent of each desalted peptide samples were analyzed by LC/MS/MS on nano-LC-Orbitrap Fusion Lumos-IC in FTFT (Thermo Fisher Scientific) interfaced with the nano-LC 1000 system, using reverse-phase chromatography with 2% to 90% acetonitrile/0.1% FA gradient over 87 min: ramp from 2% to 8% acetonitrile over 1 min, from 8% acetonitrile to 25% acetonitrile over 1 to 61 min, from 25% to 45% acetonitrile over 61 to 81 min, from 45% to 100% acetonitrile from 81 to 86 min at 300 nl/min, on 75 μm × 150 mm ProntoSIL-120-5-C18 H column 3 μm, 120 Å (BISCHOFF). Eluted peptides were sprayed into an Orbitrap Fusion Lumos-IC mass spectrometer through 1 μm emitter tip (New Objective) at 2.6 kV. Survey scans (Full MS) were acquired at a resolution 120K, within 370 to 1800 Da m/z, AGC target 4 × e^5^, max inject time 60 ms, using data-dependent Top 15 method with dynamic exclusion of 15 s after 1 time. Precursor ions were individually isolated in Quadrupole at 0.7 Da (no offset, fragmented (MS/MS)) using an HCD activation collision energy of 32 and analyzed at a resolution of 30K.

Peptide and fragment ion masses were extracted from the raw mass spectra in Proteome Discoverer (PD) software (v2.3, Thermo-Scientific) and PEAKS Studio Xpro (v. X, Bioinformatics Solution Inc) and searched using Mascot (v2.6.2, Matrix Science) against three databases containing the human NEDD4L FL WT (Uniprot: Q96PU5), human Sodium channel protein type 5 subunit alpha (Uniprot: Q14524) and *Homo sapiens* WT ubiquitin C (Uniprot: L8B196). Specific search parameters were as follows: precursor s/n 1.5, mass tolerance 5 ppm, fragment mass tolerance 0.01 Da, Lys ubiquitination (GlyGly), Met oxidation, Cys carbamidomethylation, Asn/Gln deamidation. Mascot files were sent to PD2.3 for PSM validation. All MS/MS spectra assigned to modified NEDD4L or ubiquitin peptides were manually inspected, and the relative abundances of the ubiquitin chain linkages and sites were determined using spectral counting.

### Differential scanning fluorimetry of NEDD4L variants

Reaction mixtures (20 μl) were set up in a white, unskirted 96-well PCR plates (Bio-Rad, MLL9651) by mixing 2 μl of purified NEDD4L variant proteins at a concentration of 1 mg/ml (final concentration 0.1 mg/ml) with 2 μl of 50× SYPRO orange dye (Invitrogen S6650) in TRIS pH 8.0, 200 mM NaCl. Plates were centrifuged for 1000*g* for 30 s after being sealed with an optical transparent film. Thermal scanning was performed from 25 to 100 °C (1 °C/min temperature gradient) using a CFX9 Connect real-time PCR instrument (Bio-Rad). Melting temperature (T_m_)/protein unfolding was calculated from the maximum value of the negative first derivative of the melt curve using CFX Manager Software (Bio-Rad).

### Mammalian cell culture

HEK293 cells (ATCC CRL-1573) were cultured in Dulbecco’s Modified Eagle Medium supplemented with 10% fetal bovine serum and 4 mM L-glutamine. Cells were tested for *mycoplasma* contamination with the PCR-based MycoDtect kit from Greiner Bio-One North America, Inc.

### Western blots

For cell lysates, approximately 0.25 × 10^6^ HEK293 cells were seeded in 6-well tissue culture dishes. Cells were transfected using Lipofectamine 2000 (Invitrogen) according to the manufacturer’s instructions with 1 μg/ml of full-length Na_V_1.5 pcDNA3.1 and 0.5 μg/ml of full-length NEDD4L pcDNA3.1 and were studied 24 h posttransfection. The total amount of DNA for all transfections was kept constant. Cells were lysed directly in the well with lysis buffer heated to 55 °C (62.5 mM Tris–HCl pH 7.0, 10% glycerol, 2% SDS, 0.005% bromophenol blue, 2.5% BME). Whole cell lysates were loaded on Any-kD gels (Bio-Rad) and transferred to a PVDF membrane using a Trans-Blot Turbo Transfer System (Bio-Rad) for 10 min. PVDF membranes were blocked and immunostained as described below.

Protein samples from the *in vitro* ubiquitination assays were loaded on a 12% SDS-PAGE gel and transferred to a PVDF membrane using a Power Blotter dry-blotting system (Thermo Fisher Scientific) for 10 min. Revert 700 total protein stains (LiCOR 926-11011) were performed according to the manufacturers’ instructions. The membranes were blocked with Intercept (TBS) Blocking buffer (LiCOR) for 1 h at room temperature. Anti-NEDD4 antibody (CST #2740 1:2000 dilution for *in vitro* assays) or anti-NEDD4L (ABclonal #A8085 1:1000 in 5% BSA for cell lysates) was diluted in 0.05% TBS-Tween-20 (TBS/T), added to the membranes, and incubated at 4 °C overnight. After this, anti-ubiquitin (SCBT sc-8017 1:2000 dilution), anti-K48 Ub (CST #4289 1:2000 dilution), or anti-K63 Ub (Millipore Sigma 05-1308 1:2000 dilution), or anti-GAPDH (HyTest #5G4 1:2000 dilution in 5% BSA) was added to the 0.05% TBS-Tween-20–primary antibody solution and incubated at room temperature for 1 h. The membranes were then washed with TBS/T 4 × 5 min and probed with IRDye anti-mouse 800 (ubiquitin, GAPDH) and anti-rabbit 680 (NEDD4, NEDD4L) secondary antibodies at 1:10,000 dilution. The bands were detected on a LiCOR Odyssey CLx. All antibodies were validated by staining a negative control. All assays were repeated on at least two independent occasions with replicates revealing similar results to the data in the figures.

### Quantification and statistical analysis

All the *in vitro* ubiquitination assays and Western blots were performed at least twice and given similar results. The bands were quantified using ImageJ software (version 1.53a, National Institute of Health).

### Immunofluorescent staining and confocal microscopy

Approximately 0.25 × 10^6^ HEK293 cells were seeded onto glass coverslips coated with 50 μg/ml Poly-D-Lysine (Gibco, prepared according to the manufacturer’s instructions) in 6-well tissue culture dishes. Cells were transfected using Lipofectamine 2000 (Invitrogen, according to the manufacturer’s instructions) with 1 μg/ml of full-length Na_V_1.5 pcDNA3.1 and 0.5 μg/ml of full-length NEDD4L pcDNA3.1 and were studied 24 to 72 h posttransfection. The total amount of DNA for all transfections was kept constant. Cells were fixed in 4% paraformaldehyde for 20 min at room temp. Cells were washed 3 × 10 min with PBS and blocked in 1% BSA, 5% normal goat serum, 5% normal donkey serum in 0.05% PBS-Tween-20 at room temperature shaking gently for 2 h. Cells were incubated in primary antibody in 5% BSA in 0.05% PBS-Tween-20 (anti-Na_V_1.5 Millipore AB5493 1:500 dilution and anti-NEDD4L Cell Signaling Technology #4013 1:200 dilution) at 4 °C overnight. Cells were washed 3 × 10 min with 0.05% PBS-Tween-20 at room temperature shaking gently. Cells were stained with secondary antibody (AF-488 anti-rabbit at 1:300 dilution), Phalloidin-iFlour647 dye (Abcam ab176759, 1:1000 dilution) to stain actin filaments, and Hoescht 33342 to stain DNA (Sigma Aldrich, 1:1000 dilution) in 0.05% PBS-Tween-20 for 90 min at room temperature, shaking gently, and protected from light. Coverslips with fixed cells were then washed 3 × 10 min with 0.05% PBS-Tween-20 at room temperature shaking gently before being mounted with Fluoro-Gel Mounting Medium with TES Buffer (Electron Microscopy Sciences 50-246-94). All antibodies were validated by staining a negative control. Slides were imaged on an Olympus FV3000RS confocal microscope and processed with NIH ImageJ software.

### Design of nanobodies that modulate Navs

NanoMaNs were design by cloning the coding sequence of high affinity channel-targeted nanobody Nb17 or Nb82 (44) fused to a cargo (catalytic domain of NEDD4L E3 ligase, aa 640–975) into pcDNA3.1 using BstXI and XbaI.

### Electrophysiology

HEK293 cells were isolated and seeded in 10 cm tissue culture dishes. Cells were transfected using a calcium phosphate method (49) with 5 μg full-length Na_V_1.5 pcDNA3.1 and 2.5 μg pcDNA3.1 NEDD4Lvariants as well as 3 μg SV40 T-antigen to enhance expression and 5 μg YFP to detect transfected cells. For each condition, we obtained multiple cells from three independent transfections. Glass pipettes (BF150-86-10, Sutter Instruments) were pulled with a horizontal puller (P-97; Sutter Instruments Company) and fire polished (Microforge, Narishige) to have 1 to 3 MΩ resistance. The bath solution contained the following: 140 mM NaCl, 1 mM CaCl_2_, 10 mM Hepes pH 7.4 adjusted with NaOH and at 290 mOsm adjusted with glucose. The pipet solution contained the following (in millimolars): CsMeSO_3_, 114; CsCl, 5; MgCl_2_, 1; MgATP, 4; Hepes (pH 7.4), 10; and BAPTA (1,2-bis(o-aminophenoxy)ethane-N,N,N′,N′-tetraacetic acid), 10; at 290 mOsm adjusted with glucose. Recordings were low-pass filtered at 2 kHz and sampled at 10 kHz with P/8 leak subtraction and 70% series resistance and capacitance compensation. Whole-cell I_Na_ recordings were obtained 24 h posttransfection at room temperature (∼25 °C) with an Axopatch 200B amplifier (Axon Instruments). The holding potential was −120 mV and steps of 10 mV from −90 mV to +50 mV were held for 10 ms to evaluate I_Na_. Data acquisition utilized an ITC-18 (Instrutech) data acquisition unit controlled by custom MATLAB software (Mathworks). The voltage-dependence of activation was determined by fitting current-voltage relationships for each individual cell with the following equation using least squares minimization:

$$I\left(V\right)=G\cdot 1/{\left(1+\exp\left(-\left(V-V_{1/2,act}\right)/SF\right)\right)}^{4}\cdot \left(V-V_{rev}\right)$$

### Statistical tests

Peak current density electrophysiology data were analyzed for statistical significance using a one-way ANOVA with Tukey’s multiple comparison (GraphPad Prism). For multiple testing, data were tested for normal distribution and we used a false discovery rate of 0.05. All graphs show the mean ± SD and were generated using GraphPad Prism.

## Data availability

All data have been provided in this manuscript. All mass spectrometry raw data have been deposited at iProX Consortium and are publicly available as of the date of publication. This paper does not report original code.

## Supporting information

This article contains supporting information.

## Conflict of interest

The authors declare no conflicts of interest in regards to this manuscript. S. B. G. is a cofounder and equity holder in the company Advanced Molecular Sciences, LLC. S. B. G. has been or is a consultant for Scorpion Therapeutics and Xinthera. P. A. C. has been a consultant for Scorpion Therapeutics.

## Supporting information

Supporting Figure S1 **The C2 domain and 1,2-linker synergistically increase NEDD4L activity.***A*, *in vitro* ubiquitination assay of the FL^NEDD4L^ and the catalytically inactive CS^NEDD4L^ (Cys942Ser) proteins. Samples were quenched with reducing loading buffer at the indicated time points. The amount of unmodified FL^NEDD4L^ and CS^NEDD4L^ proteins, quantified by a densitometry analysis as a function of time, is shown as a percentage averaging all replicates. The average percentages ± SD for FL^NEDD4L^ and CS^NEDD4L^ are as follows (%): 100, 46 ± 5, 34 ± 2; 100, 94 ± 4, 90 ± 5, respectively. All the assays were repeated at least twice (N ≥ 2). *B*, extended time course of an *in vitro* ubiquitination activity of 1,2-linker^NEDD4L^, ΔC2^NEDD4L^, and W2-W3-W4-HECT^NEDD4L^. Equal amounts of samples were taken at the indicated time points and quenched with reducing loading buffer. The amount of unmodified NEDD4L proteins, quantified by a densitometry analysis as a function of time, is shown as a percentage averaging all replicates. The average percentages ± SD for NEDD4L proteins are as follows (%): 100, 64 ± 15, 56 ± 12, 38 ± 6, 23 ± 4, 16 ± 3; 100, 77 ± 11, 71 ± 6, 45 ± 10, 34 ± 18, 22 ± 5; 100, 80 ± 15, 60 ± 13, 33 ± 17, 8 ± 3, 4 ± 3. All the assays were repeated at least twice (N ≥ 2).

Supporting Figure S2 **The C2 domain and 1,2-linker inhibit E2-E3 transthioesterification.***A*, schematic of the E2-E3 transthioesterification assay. The E1 enzyme, E2 (UbcH5c) enzyme, and fluorescently labeled ubiquitin (FAM-Ub) were incubated for 40 min at RT to generate a E2∼Ub thioester intermediate. The reaction was quenched with EDTA and then the E3 ligases were added. Formation of the E3∼Ub intermediate and disappearance of E2∼Ub was monitored. *B*, fluorescent imaging of SDS-PAGE gel monitoring the natural transthioesterification of E2∼Ub in the absence of an E3 ligase. Samples were quenched with both non-reducing 2× and reducing loading buffer at 0, 5, 10, and 15 min. All the assays were repeated at least twice (N ≥ 2). *C*, schematic representation of full-length WWP2 (FL^WWP2^ aa 1–870) and Δ2,3-linker^WWP2^ (aa 1–361/394–870). The C2 domain is shown in *green*; WW domains in *light blue*; HECT domain shown in *orange* (N-lobe) and *pink* (C-lobe) with the active site cysteine residue highlighted in a *yellow pentagon*. *D*, schematic representation of full-length NEDD4, isoform 4 (FL^NEDD4^ aa 1–9000), and Δ1,2-linker^NEDD4^ (aa 1–224/245–900). The domain scheme is colored as in (*A*). *E*, fluorescent imaging of SDS-PAGE monitoring the transfer of ubiquitin from the E2 enzyme by FL^WWP2^ and Δ2,3-linker^WWP2^. Samples were quenched with nonreducing 2× loading buffer at 0, 3, 10, and 15 min for FL^WWP2^ and 0, 1, 3, and 5 min for Δ2,3-linker^WWP2^. The amount of unmodified E2∼Ub, quantified by a densitometry analysis as a function of time, is shown as a percentage averaging all replicates. The average percentages ± SD for the E2∼Ub bands are as follows (%): 100, 99 ± 9, 64 ± 10, 16 ± 9, 100, 66 ± 5, 10 ± 1, 1 ± 1. All the assays were repeated at least twice (N ≥ 2). *F*, fluorescent imaging of SDS-PAGE monitoring the transfer of ubiquitin from the E2 enzyme by FL^WWP2^ and Δ2,3-linker^WWP2^. Samples were quenched with reducing (DTT-treated) 2× loading buffer at 0, 3, 10, and 15 min for FL^WWP2^ and 0, 1, 3, and 5 min for Δ2,3-linker^WWP2^. All assays were repeated at least twice with good reproducibility (n = 2). *G*, fluorescent imaging of SDS-PAGE monitoring the transfer of ubiquitin from the E2 enzyme by FL^NEDD4^ and Δ1,2-linker^NEDD4^. Samples were quenched with nonreducing 2× loading buffer at 0, 10, 30, and 60 min for FL^NEDD4^ and 0, 1, 3, and 5 min for Δ1,2-linker^NEDD4^. The amount of unmodified E2∼Ub, quantified by a densitometry analysis as a function of time, is shown as a percentage averaging all replicates. The average percentages ± SD for the E2∼Ub bands are as follows (%): 100, 73 ± 15, 59 ± 18, 26 ± 5; 100, 36 ± 6, 20 ± 1, 11 ± 1. All the assays were repeated at least twice (N ≥ 2). *H*, fluorescent imaging of SDS-PAGE monitoring the transfer of ubiquitin from the E2 enzyme by FL^NEDD4^ and Δ1,2-linker^NEDD4^. Samples were quenched with reducing (DTT-treated) 2× loading buffer at 0, 10, 30, and 60 min for FL^NEDD4^ and 0, 1, 3, and 5 min for Δ1,2-linker^NEDD4^. All the assays were repeated at least twice (N ≥ 2).

Supporting Figure S3 **E2-E3 transthioesterification assays modify NEDD4 family E3 ligaes through lysine residues.***A*, fluorescent imaging of SDS-PAGE gels monitoring the transfer of ubiquitin from the E2 enzyme by FL^NEDD4L^, ΔC2^NEDD4L^, Δ1,2-linker^NEDD4L^, and Δ2,3-linker^NEDD4L^. Samples were quenched with non-reducing 2× loading buffer at 0, 5, 10, and 15 min. The amount of unmodified E2∼Ub, quantified by a densitometry analysis as a function of time, is shown as a percentage averaging all replicates. The average percentages ± SD for the E2∼Ub bands are as follows (%): 100, 19 ± 9, 17 ± 8, 14 ± 5; 100, 18 ± 18, 10 ± 12, 8 ± 10; 100, 9 ± 5, 7 ± 3, 5 ± 3; 100, 26 ± 11, 18 ± 9, 14 ± 9. All the assays were repeated at least twice (N ≥ 2). *B*, fluorescent imaging of SDS-PAGE monitoring the transfer of ubiquitin from the E2 enzyme by FL^NEDD4L^, ΔC2^NEDD4L^ , Δ1,2-linker^NEDD4L^, and Δ2,3-linker^NEDD4L^. Samples were quenched with reducing (DTT-treated) 2× loading buffer at 0, 5, 10, and 15 min. All the assays were repeated at least twice (N ≥ 2).

Supporting Figure S4 **Ubiquitin chain linkage site-specificity of NEDD4L.***A*, ubiquitin sequence coverage after band excision from the *in vitro* assay. Identified peptides are highlighted in *shaded*, *boxed gray regions*. *Bold*, *red letters* with a *yellow square* above them are identified Lysine sites of ubiquitination. *B*, ubiquitin lysine chain linkage of the FL^NEDD4L^*in vitro* autoubiquitination assay was analyzed by LC/MS/MS. The peptide spectrum matches of each ubiquitin Lysine residue seen with a Gly-Gly modification is represented as a pie graph. *C*, representative MS/MS spectrum and sequence coverage of the peptide (TLSDYNIQkESTLHLVLR) containing a Gly-Gly modification on ubiquitin Lys63. Lower case k indicates Gly-Gly modification. *D*, *in vitro* ubiquitination assays of FL^NEDD4L^ in the presence of Ub^WT^, Ub^K48R^, or Ub^K63R^. Samples were quenched with reducing 2× SDS-PAGE loading buffer at 0, 10, 30, and 90 min and the gel was stained with colloidal Coomassie blue stain. The amount of unmodified FL^NEDD4L^ protein, quantified by a densitometry analysis as a function of time, is shown as a percentage averaging all replicates. The average percentages ± SD for FL^NEDD4L^ in the presence of Ub mutants are as follows (%): 100, 69 ± 2, 58 ± 10, 48 ± 14; 100, 87 ± 6, 60 ± 6, 40 ± 8; 100, 21 ± 5, 5 ± 3, 2 ± 1. All the assays were repeated at least twice (N ≥ 2). *E*, densitometry analysis, represented as a bar graph, of the average percentages of unmodified FL^NEDD4L^ clustered by Ub mutant and taken at the indicated time points of (*E*).

Supporting Figure S5 **Na**_**V**_**1.5 ubiquitination by NEDD4L on Na**_**V**_**1.5 DI-DII linker requires CTerm PY motif.***A*, schematic representation of Na_V_1.5 topology. The alpha subunit of Na_V_1.5 contains four domains (DI-DIV; *green*) that each contain six transmembrane segments (labeled 1–6). Segments 5 and 6 (*light green*) form the channel pore while segments 4 (*medium green*) contain positively charged residues that confer the channel’s voltage-sensitivity. The cytoplasmic interdomain linker between DI and DII is shown in *purple*. The CTerm is shown with *dark blue* cylinders representing alpha-helices as visible in the crystallographic structure that terminates at residue 1929 (PDB ID 4OVN; Fig. 7*A*). The canonical NEDD4L binding motif PPSY is indicated in the *light blue box* at residues 1974 to 1977. The accessory beta subunit is shown in *yellow*. *B*, time course of the *in vitro* ubiquitination assay with FL^NEDD4L^ in the absence and presence of the Na_V_1.5^CTerm^ substrate. Equal amounts of samples were taken at 0, 30, 60, 180 min and quenched with reducing loading buffer. The SDS-PAGE was stained with colloidal Coomassie blue stain. The amount of unmodified Na_V_1.5^CTerm^ protein, quantified by a densitometry analysis as a function of time, is shown as a percentage averaging all replicates. The average ± SD for Na_V_1.5^CTerm^ protein in the presence of NEDD4L enzyme are as follows (%): 100, 98 ± 2, 96 ± 2, 99 ± 4. All the assays were repeated at least twice (N ≥ 2). *C*, densitometry analysis, represented as a bar graph, of the unmodified Na_V_1.5^CTerm^ taken at the indicated time points. Error bars represent SD. *D*, *in vitro* ubiquitination time course of FL^NEDD4L^ in the absence and presence of Na_V_1.5^DI-DII+PPSY^ substrate. Equal amounts of samples were taken at the indicated time points and quenched with reducing loading buffer. The SDS-PAGE was stained with colloidal Coomassie blue stain. The amount of unmodified Na_V_1.5 ^DI-DII+PPSY^ protein, quantified by a densitometry analysis as a function of time, is shown as a percentage averaging all replicates. The average ± SD for Na_V_1.5 ^DI-DII^ variant proteins in the presence of NEDD4L enzyme are as follows (%): 100, 62 ± 3, 49 ± 2, 24 ± 1. All the assays were repeated at least twice (N ≥ 2). Purified Na_V_1.5^DI-DII+PPSY^ chimera alone in lane 6 (labeled P). *E*, densitometry analysis, represented as a bar graph, of the unmodified Na_V_1.5 ^DI-DII^ taken at the indicated time points. Error bars represent SD. *F*–*H*, representative MS/MS spectrum and sequence coverage of the Na_V_1.5^DI-DII-linker^ peptides (FGEAmEmLkK) containing a Gly-Gly modification on Lys442, (FQEAmEmLKkEHEALTIR) containing a Gly-Gly modification on Na_V_1.5^DI-DII-linker^ Lys443, and (mSSGTEEcGEDRLPkSDSEDGPR) containing a Gly-Gly modification on Na_V_1.5^DI-DII-linker^ Lys496. Lower case k indicates a Gly-Gly modification and lower case m indicates an oxidized methionine.

Supporting Figure S6 **Ubiquitin chain linkage site-specifictiy of modified Na**_**v**_**1.5 shows primarily Lys63-linked chains.***A*, ubiquitin sequence coverage after di-Ub-Na_V_1.5^DI-DII+PPSY^ band excision from the *in vitro* assay. Identified peptides are highlighted in *shaded*, *boxed gray regions*. *Bold*, *red letters* with a *yellow square* above them are identified Lysine sites of ubiquitination. *B*, ubiquitin lysine chain linkage of Na_V_1.5^DI-DII+PPSY^ modified by FL^NEDD4LL^*in vitro* ubiquitination assay was analyzed by LC/MS/MS. The peptide spectrum matches of each ubiquitin Lysine residue seen with a Gly-Gly modification is represented as a pie graph. *C*, representative MS/MS spectrum and sequence coverage of the peptide (TLsDYNIQkESTLHLVLR) containing a Gly-Gly modification on ubiquitin Lys63 and (*D*) the peptide (LIFAGkQLEDGR) containing a Gly-Gly modification on ubiquitin Lys48. Lower case k indicates a Gly-Gly modification and lower case m indicates an oxidized methionine. *E*, fluorescent Western blot analysis of the *in vitro* FL^NEDD4L^ autoubiquitination and Na_V_1.5^DI-DII+PPSY^ substrate assays. Immunofluorescent staining was performed using an anti-K63-linked Ub (*green*) antibody. The *purple arrow* indicates unmodified Na_V_1.5^DI-DII+PPSY^ and the *pink arrow* indicates FL^NEDD4L^ as visible in (*F*) total protein stain of the same PVDF membrane. *G*, fluorescent Western blot analysis of the *in vitro* FL^NEDD4L^ autoubiquitination and Na_V_1.5^DI-DII+PPSY^ substrate assays. Immunofluorescent staining was performed using an anti-K48-linked Ub (*green*) antibody. The *purple arrow* indicates unmodified Na_V_1.5^DI-DII+PPSY^ and the *pink arrow* indicates FL^NEDD4L^ as visible in (*H*) total protein stain of the same PVDF membrane.

Supporting Figure S7 **Δ1,2-linker**^**NEDD4L**^**shows expedited ubiquitination of Na**_**V**_**1.5**^**DI-DII+PPSY**^***in vitro*.***A*, *in vitro* ubiquitination assays of FL^NEDD4L^, ΔC2^NEDD4L^, Δ1,2-linker^NEDD4L^, and Δ2,3-linker^NEDD4L^ in the presence of Na_V_1.5^DI-DII+PPSY^ substrate. Samples were quenched with reducing 2× SDS-PAGE loading buffer at 0, 30, 60, and 180 min and the gels were stained with colloidal Coomassie blue stain. The amount of unmodified Na_V_1.5 ^DI-DII+PPSY^ protein, quantified by a densitometry analysis as a function of time, is shown as a percentage averaging all replicates. The average ± SD for Na_V_1.5 ^DI-DII^ variant proteins in the presence of NEDD4L enzyme variants are as follows (%): 100, 60 ± 0, 48 ± 1, 25 ± 5; 100, 73 ± 2, 61 ± 2, 42 ± 8; 100, 57 ± 2, 40 ± 5, 26 ± 7; 100, 74 ± 2, 60 ± 2, 30 ± 2. All the assays were repeated at least twice (N ≥ 2). *B*, fluorescent Western blot analysis of the *in vitro* assays in (*A*). Immunofluorescent staining was performed using an anti-Ub (*red*) antibody. The *purple bracket* indicates the location of Na_V_1.5^DI-DII+PPSY^ and the *black bracket* indicates FL^NEDD4L^. *C*, dot plot shows voltage-dependence of activation (*V*_1/2,act_) obtained by fitting current-voltage relations. Minimal change in *V*_1/2,act_ was observed upon co-expression of NEDD4L variants. Mean ± SD (*D*) V_1/2,act_ was unperturbed for Na_V_1.5 PPSY/AASA mutant. *E*, V_1/2,act_ was unperturbed for Na_V_1.5 K442/443/496-R mutant.
