# Supplementary figures and images for: NEDD4L intramolecular interactions regulate its auto and substrate NaV1.5 ubiquitination
Source: J Biol Chem. 2024 Feb 2;300(3):105715. doi: 10.1016/j.jbc.2024.105715 (PMC10933555; doi:10.1016/j.jbc.2024.105715)

**A**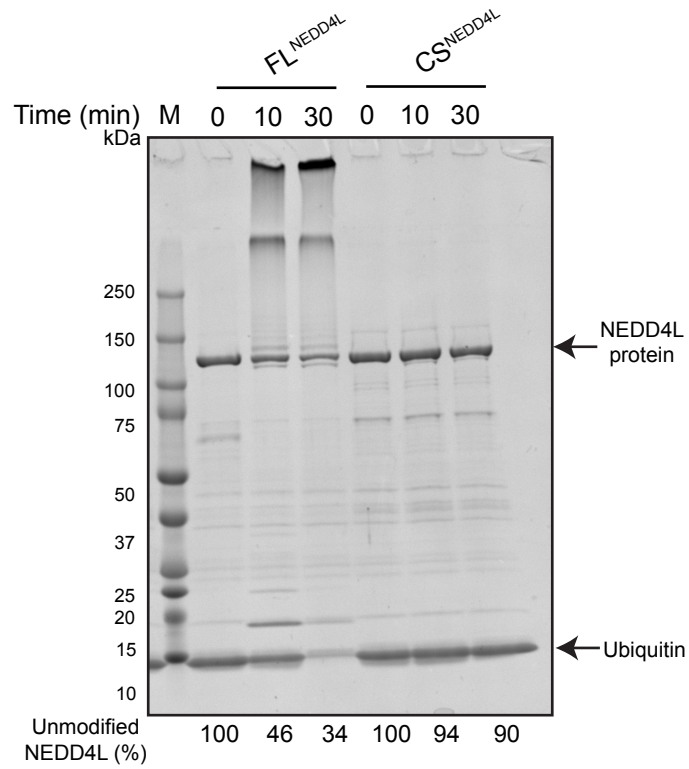**B**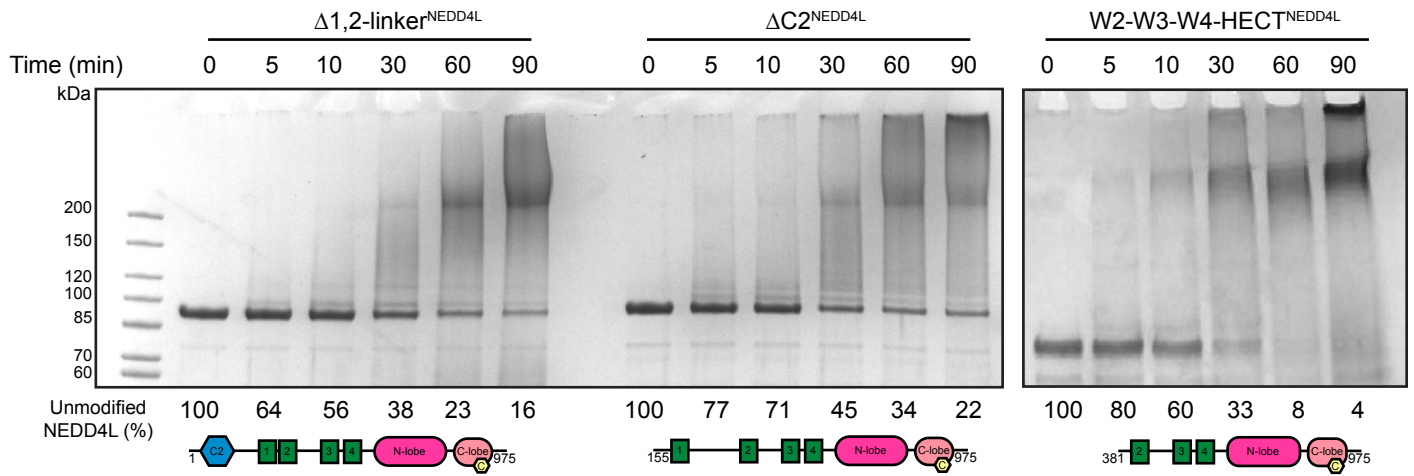

Supplement: Supporting Figure S1 — The C2 domain and 1,2-linker synergistically increase NEDD4L activity.A, in vitro ubiquitination assay of the FLNEDD4L and the catalytically inactive CSNEDD4L (Cys942Ser) proteins. Samples were quenched with reducing loading buffer at the indicated time points. The amount of unmodified FLNEDD4L and CSNEDD4L proteins, quantified by a densitometry analysis as a function of time, is shown as a percentage averaging all replicates. The average percentages ± SD for FLNEDD4L and CSNEDD4L are as follows (%): 100, 46 ± 5, 34 ± 2; 100, 94 ± 4, 90 ± 5, respectively. All the assays were repeated at least twice (N ≥ 2). B, extended time course of an in vitro ubiquitination activity of 1,2-linkerNEDD4L, ΔC2NEDD4L, and W2-W3-W4-HECTNEDD4L. Equal amounts of samples were taken at the indicated time points and quenched with reducing loading buffer. The amount of unmodified NEDD4L proteins, quantified by a densitometry analysis as a function of time, is shown as a percentage averaging all replicates. The average percentages ± SD for NEDD4L proteins are as follows (%): 100, 64 ± 15, 56 ± 12, 38 ± 6, 23 ± 4, 16 ± 3; 100, 77 ± 11, 71 ± 6, 45 ± 10, 34 ± 18, 22 ± 5; 100, 80 ± 15, 60 ± 13, 33 ± 17, 8 ± 3, 4 ± 3. All the assays were repeated at least twice (N ≥ 2). [file mmc1.pdf]

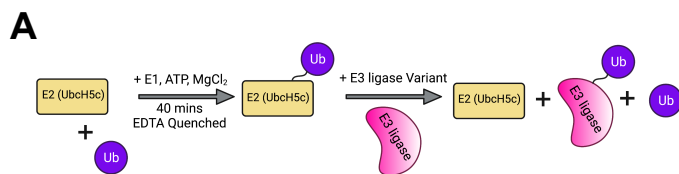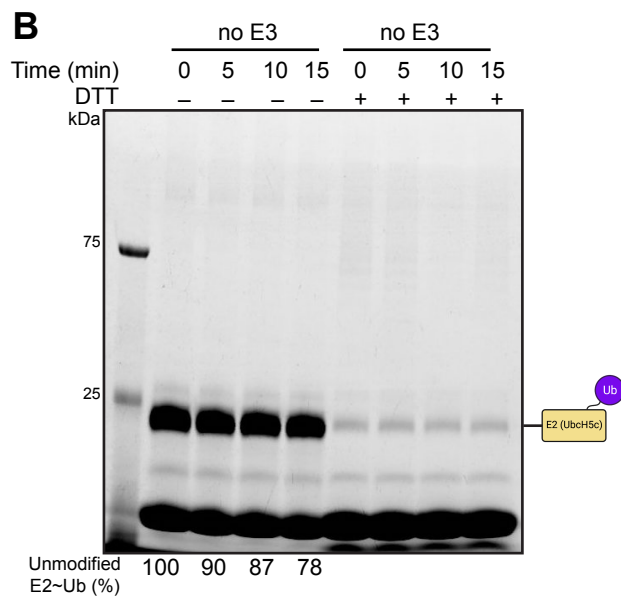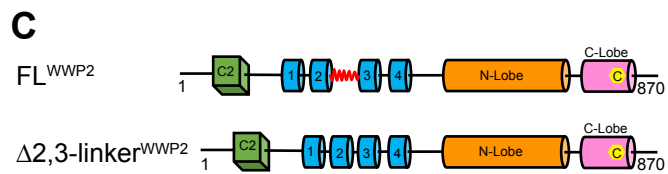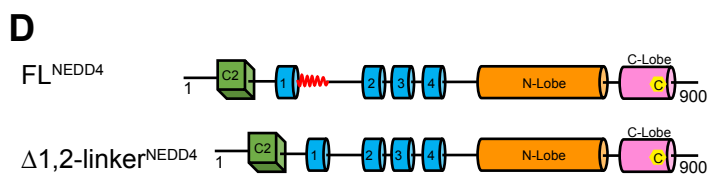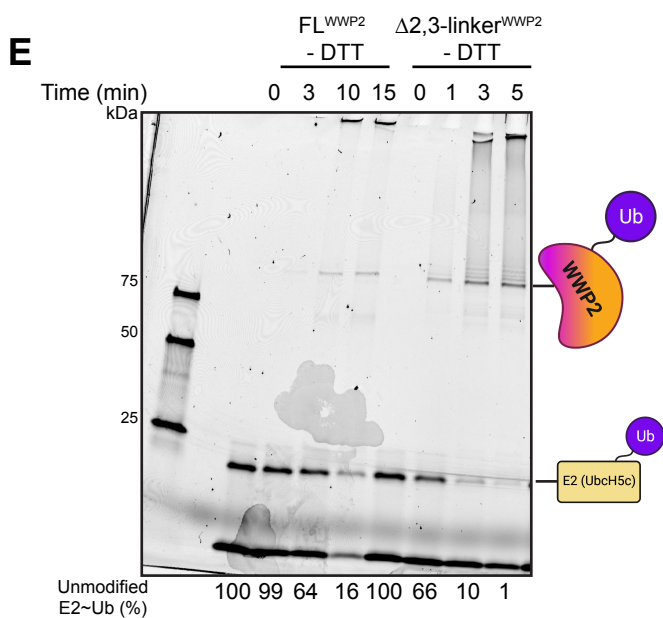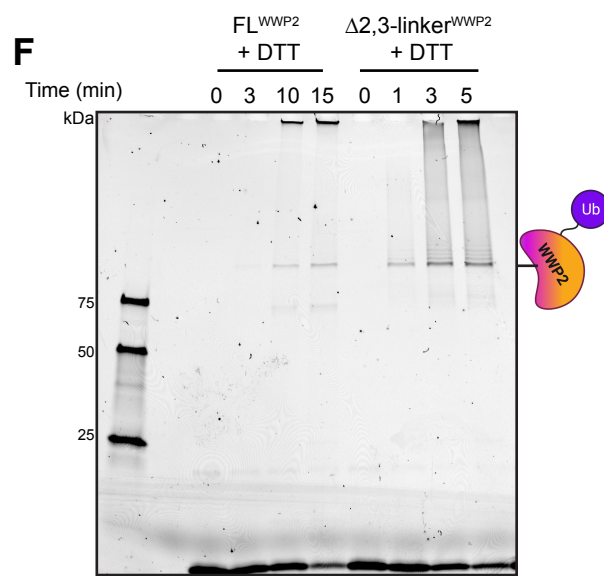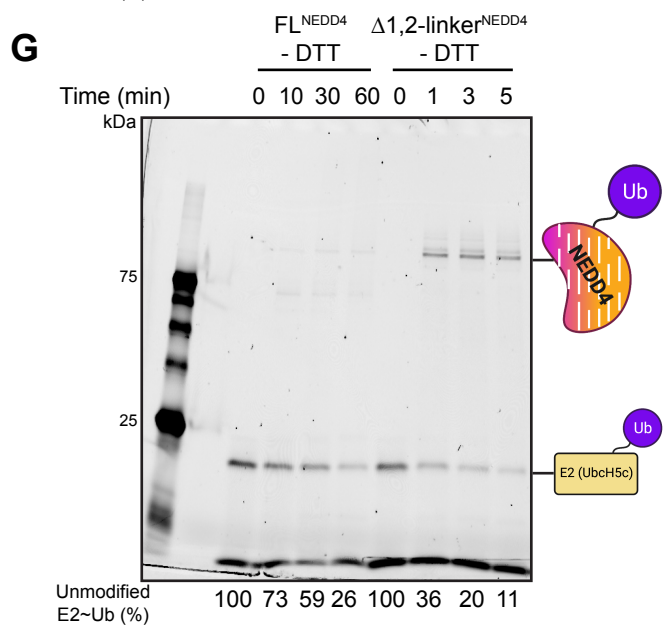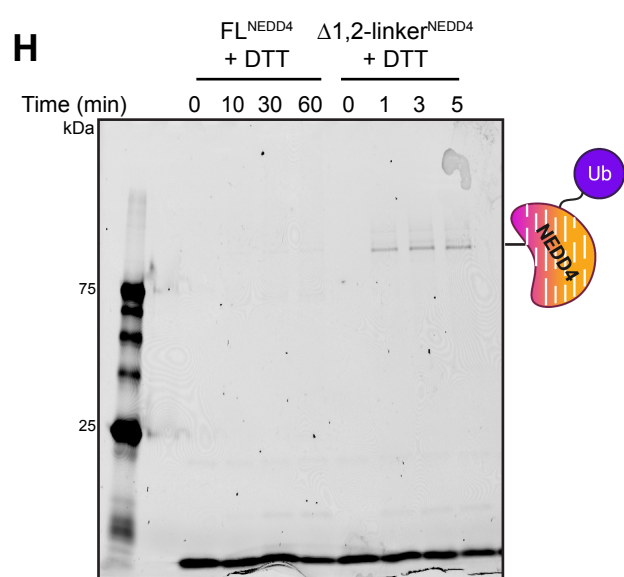

Supplement: Supporting Figure S2 — The C2 domain and 1,2-linker inhibit E2-E3 transthioesterification.A, schematic of the E2-E3 transthioesterification assay. The E1 enzyme, E2 (UbcH5c) enzyme, and fluorescently labeled ubiquitin (FAM-Ub) were incubated for 40 min at RT to generate a E2∼Ub thioester intermediate. The reaction was quenched with EDTA and then the E3 ligases were added. Formation of the E3∼Ub intermediate and disappearance of E2∼Ub was monitored. B, fluorescent imaging of SDS-PAGE gel monitoring the natural transthioesterification of E2∼Ub in the absence of an E3 ligase. Samples were quenched with both non-reducing 2× and reducing loading buffer at 0, 5, 10, and 15 min. All the assays were repeated at least twice (N ≥ 2). C, schematic representation of full-length WWP2 (FLWWP2 aa 1–870) and Δ2,3-linkerWWP2 (aa 1–361/394–870). The C2 domain is shown in green; WW domains in light blue; HECT domain shown in orange (N-lobe) and pink (C-lobe) with the active site cysteine residue highlighted in a yellow pentagon. D, schematic representation of full-length NEDD4, isoform 4 (FLNEDD4 aa 1–9000), and Δ1,2-linkerNEDD4 (aa 1–224/245–900). The domain scheme is colored as in (A). E, fluorescent imaging of SDS-PAGE monitoring the transfer of ubiquitin from the E2 enzyme by FLWWP2 and Δ2,3-linkerWWP2. Samples were quenched with nonreducing 2× loading buffer at 0, 3, 10, and 15 min for FLWWP2 and 0, 1, 3, and 5 min for Δ2,3-linkerWWP2. The amount of unmodified E2∼Ub, quantified by a densitometry analysis as a function of time, is shown as a percentage averaging all replicates. The average percentages ± SD for the E2∼Ub bands are as follows (%): 100, 99 ± 9, 64 ± 10, 16 ± 9, 100, 66 ± 5, 10 ± 1, 1 ± 1. All the assays were repeated at least twice (N ≥ 2). F, fluorescent imaging of SDS-PAGE monitoring the transfer of ubiquitin from the E2 enzyme by FLWWP2 and Δ2,3-linkerWWP2. Samples were quenched with reducing (DTT-treated) 2× loading buffer at 0, 3, 10, and 15 min for FLWWP2 and 0, 1, 3, and 5 min for Δ [file mmc2.pdf]

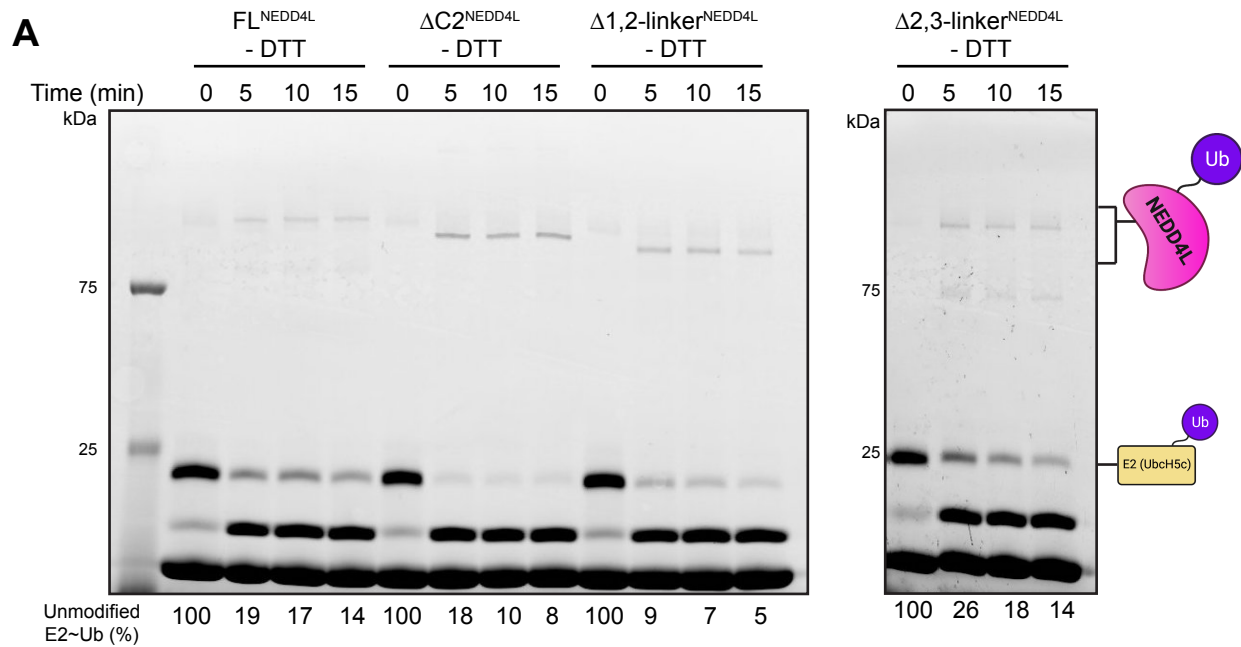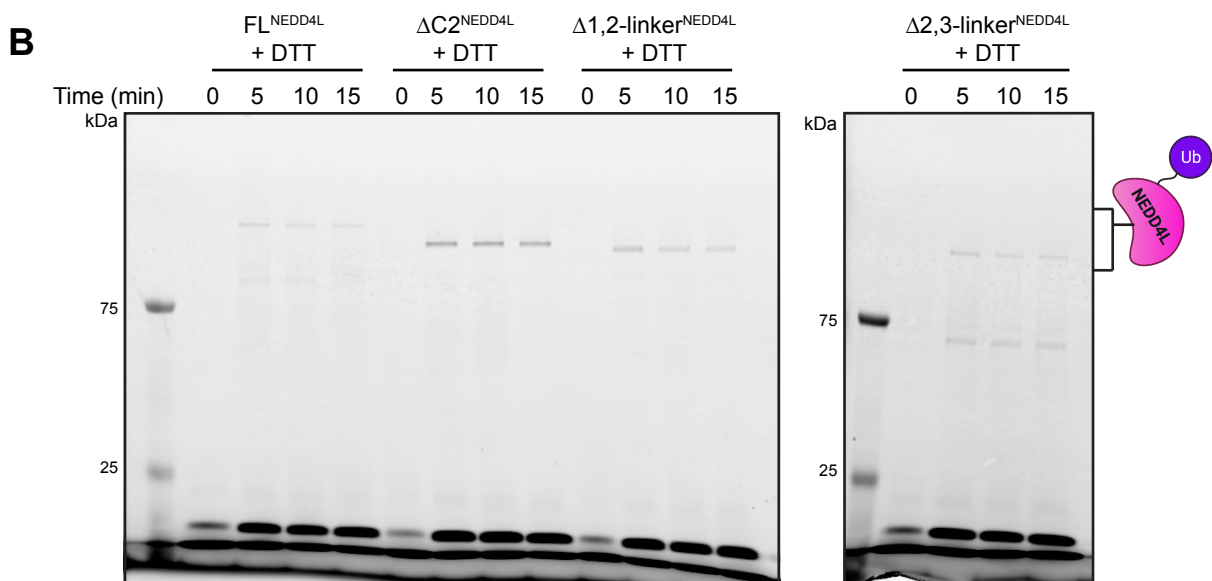

Supplement: Supporting Figure S3 — E2-E3 transthioesterification assays modify NEDD4 family E3 ligaes through lysine residues.A, fluorescent imaging of SDS-PAGE gels monitoring the transfer of ubiquitin from the E2 enzyme by FLNEDD4L, ΔC2NEDD4L, Δ1,2-linkerNEDD4L, and Δ2,3-linkerNEDD4L. Samples were quenched with non-reducing 2× loading buffer at 0, 5, 10, and 15 min. The amount of unmodified E2∼Ub, quantified by a densitometry analysis as a function of time, is shown as a percentage averaging all replicates. The average percentages ± SD for the E2∼Ub bands are as follows (%): 100, 19 ± 9, 17 ± 8, 14 ± 5; 100, 18 ± 18, 10 ± 12, 8 ± 10; 100, 9 ± 5, 7 ± 3, 5 ± 3; 100, 26 ± 11, 18 ± 9, 14 ± 9. All the assays were repeated at least twice (N ≥ 2). B, fluorescent imaging of SDS-PAGE monitoring the transfer of ubiquitin from the E2 enzyme by FLNEDD4L, ΔC2NEDD4L , Δ1,2-linkerNEDD4L, and Δ2,3-linkerNEDD4L. Samples were quenched with reducing (DTT-treated) 2× loading buffer at 0, 5, 10, and 15 min. All the assays were repeated at least twice (N ≥ 2). [file mmc3.pdf]

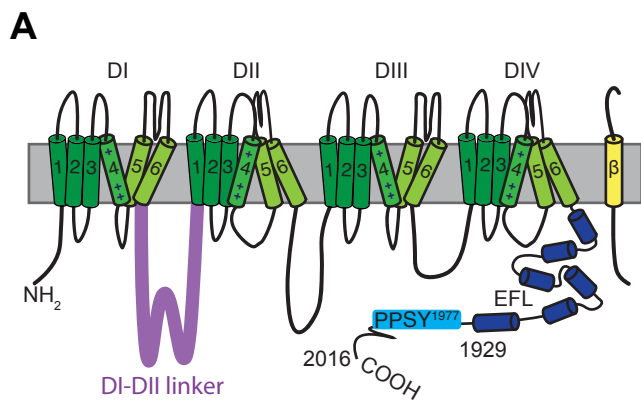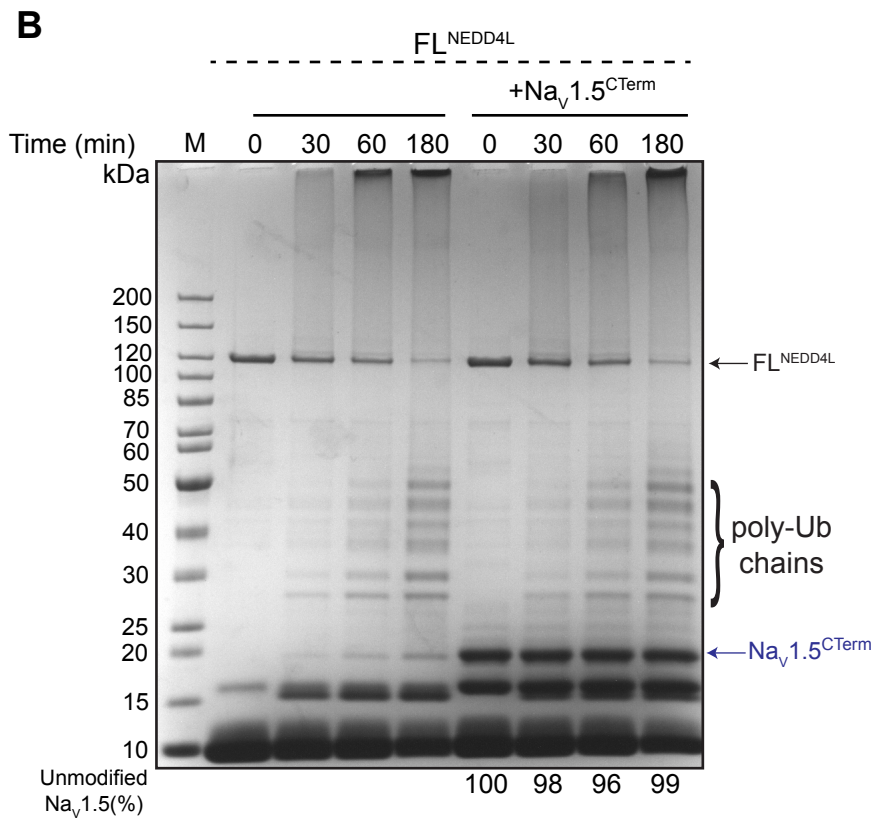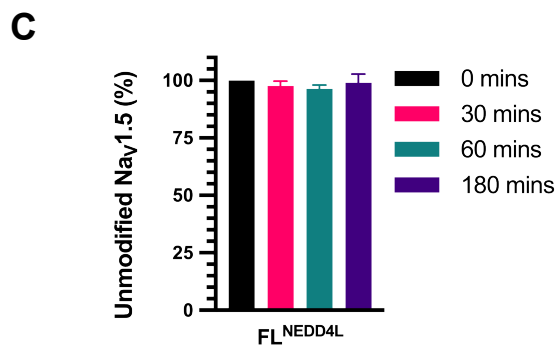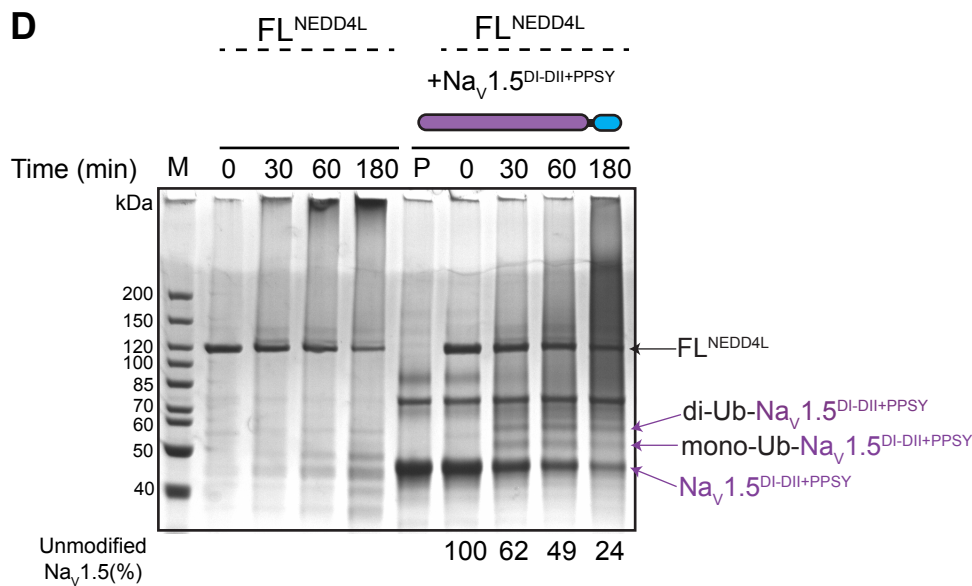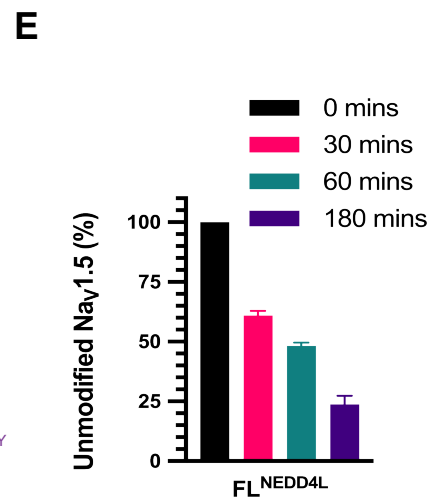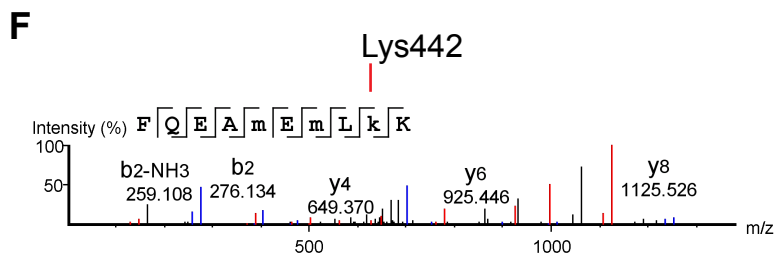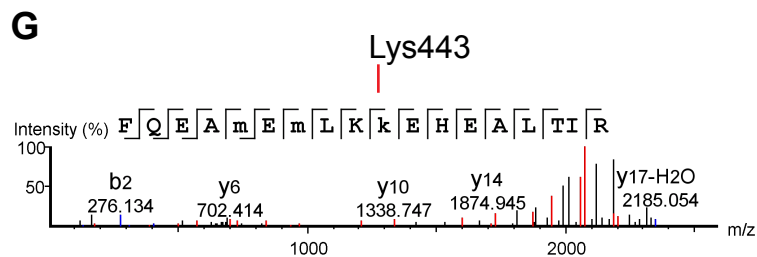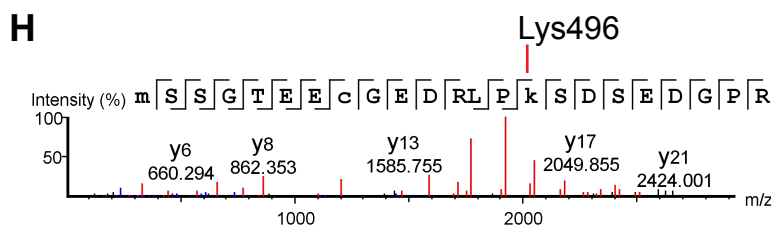

Supplement: Supporting Figure S5 — NaV1.5 ubiquitination by NEDD4L on NaV1.5 DI-DII linker requires CTerm PY motif.A, schematic representation of NaV1.5 topology. The alpha subunit of NaV1.5 contains four domains (DI-DIV; green) that each contain six transmembrane segments (labeled 1–6). Segments 5 and 6 (light green) form the channel pore while segments 4 (medium green) contain positively charged residues that confer the channel’s voltage-sensitivity. The cytoplasmic interdomain linker between DI and DII is shown in purple. The CTerm is shown with dark blue cylinders representing alpha-helices as visible in the crystallographic structure that terminates at residue 1929 (PDB ID 4OVN; Fig. 7A). The canonical NEDD4L binding motif PPSY is indicated in the light blue box at residues 1974 to 1977. The accessory beta subunit is shown in yellow. B, time course of the in vitro ubiquitination assay with FLNEDD4L in the absence and presence of the NaV1.5CTerm substrate. Equal amounts of samples were taken at 0, 30, 60, 180 min and quenched with reducing loading buffer. The SDS-PAGE was stained with colloidal Coomassie blue stain. The amount of unmodified NaV1.5CTerm protein, quantified by a densitometry analysis as a function of time, is shown as a percentage averaging all replicates. The average ± SD for NaV1.5CTerm protein in the presence of NEDD4L enzyme are as follows (%): 100, 98 ± 2, 96 ± 2, 99 ± 4. All the assays were repeated at least twice (N ≥ 2). C, densitometry analysis, represented as a bar graph, of the unmodified NaV1.5CTerm taken at the indicated time points. Error bars represent SD. D, in vitro ubiquitination time course of FLNEDD4L in the absence and presence of NaV1.5DI-DII+PPSY substrate. Equal amounts of samples were taken at the indicated time points and quenched with reducing loading buffer. The SDS-PAGE was stained with colloidal Coomassie blue stain. The amount of unmodified NaV1.5 DI-DII+PPSY protein, quantified by a densitometry analysis as a function of time, is shown as a percentag [file mmc5.pdf]

**A**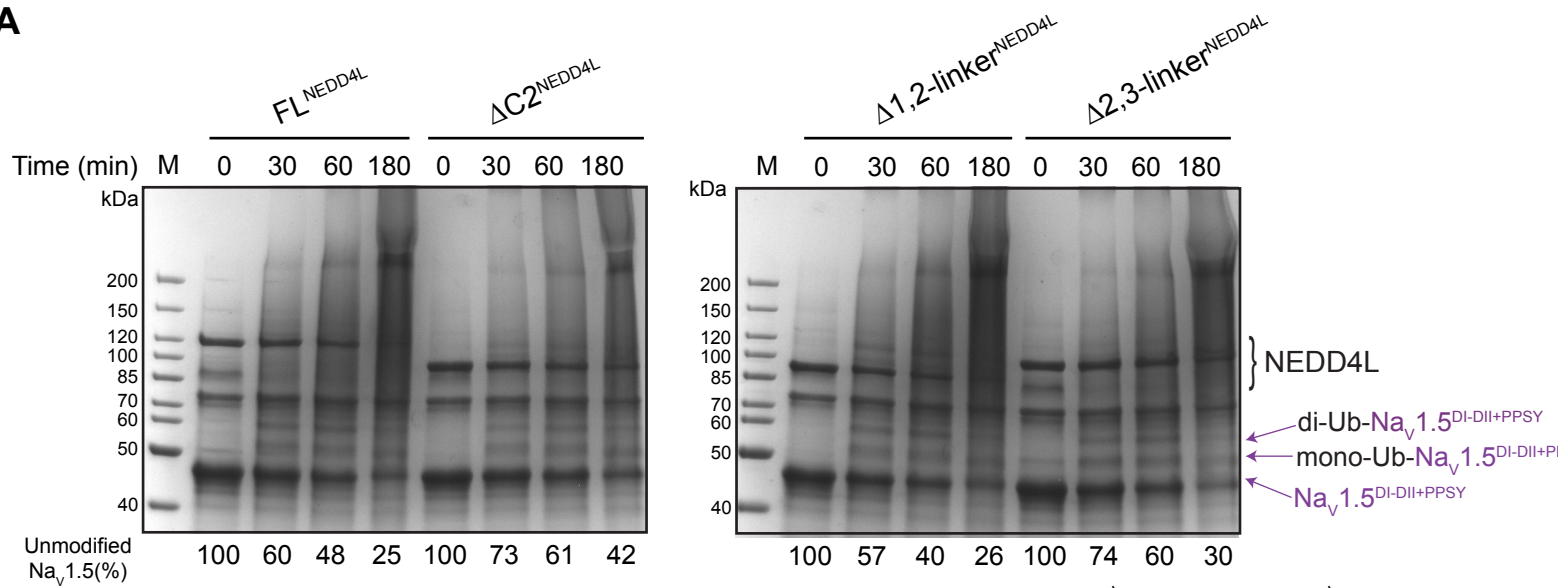**B**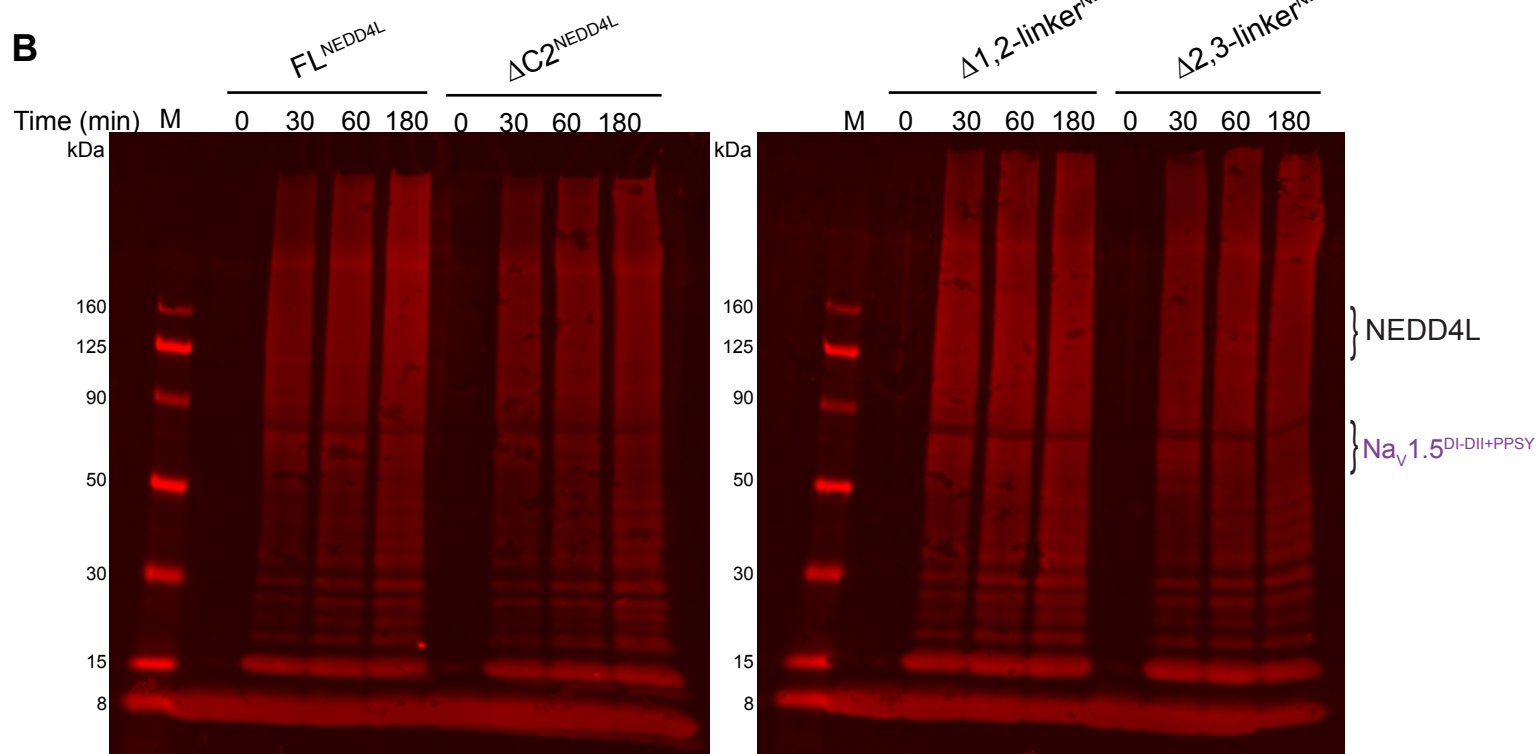**C**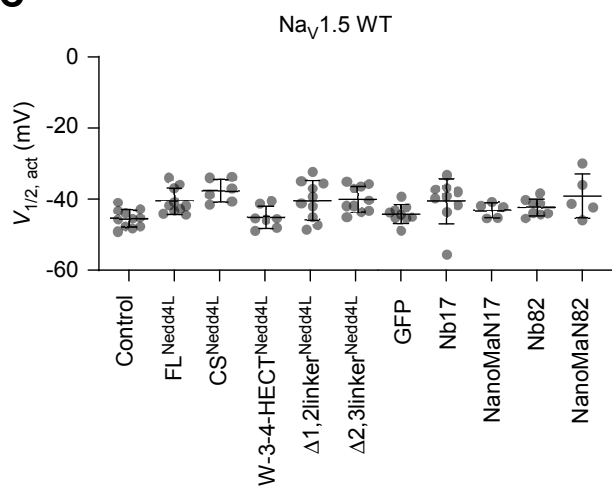**D**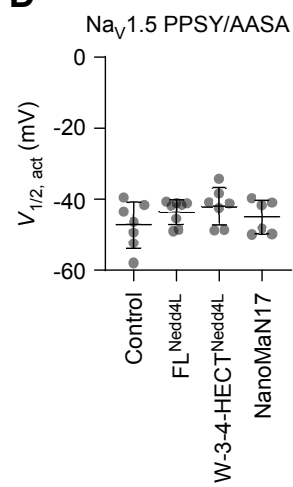**E**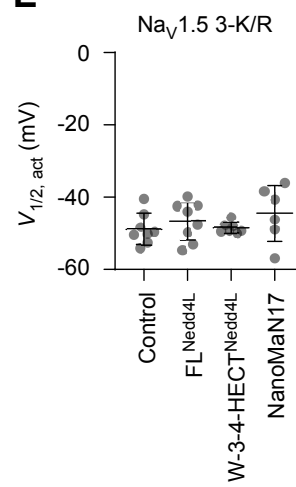

Supplement: Supporting Figure S7 — Δ1,2-linkerNEDD4Lshows expedited ubiquitination of NaV1.5DI-DII+PPSYin vitro.A, in vitro ubiquitination assays of FLNEDD4L, ΔC2NEDD4L, Δ1,2-linkerNEDD4L, and Δ2,3-linkerNEDD4L in the presence of NaV1.5DI-DII+PPSY substrate. Samples were quenched with reducing 2× SDS-PAGE loading buffer at 0, 30, 60, and 180 min and the gels were stained with colloidal Coomassie blue stain. The amount of unmodified NaV1.5 DI-DII+PPSY protein, quantified by a densitometry analysis as a function of time, is shown as a percentage averaging all replicates. The average ± SD for NaV1.5 DI-DII variant proteins in the presence of NEDD4L enzyme variants are as follows (%): 100, 60 ± 0, 48 ± 1, 25 ± 5; 100, 73 ± 2, 61 ± 2, 42 ± 8; 100, 57 ± 2, 40 ± 5, 26 ± 7; 100, 74 ± 2, 60 ± 2, 30 ± 2. All the assays were repeated at least twice (N ≥ 2). B, fluorescent Western blot analysis of the in vitro assays in (A). Immunofluorescent staining was performed using an anti-Ub (red) antibody. The purple bracket indicates the location of NaV1.5DI-DII+PPSY and the black bracket indicates FLNEDD4L. C, dot plot shows voltage-dependence of activation (V1/2,act) obtained by fitting current-voltage relations. Minimal change in V1/2,act was observed upon co-expression of NEDD4L variants. Mean ± SD (D) V1/2,act was unperturbed for NaV1.5 PPSY/AASA mutant. E, V1/2,act was unperturbed for NaV1.5 K442/443/496-R mutant. [file mmc7.pdf]
