# Supplementary material for: NEDD4L intramolecular interactions regulate its auto and substrate NaV1.5 ubiquitination
Source: J Biol Chem. 2024 Feb 2;300(3):105715. doi: 10.1016/j.jbc.2024.105715 (PMC10933555; doi:10.1016/j.jbc.2024.105715)
Supplement: Supporting Figure S4 — Ubiquitin chain linkage site-specificity of NEDD4L.A, ubiquitin sequence coverage after band excision from the in vitro assay. Identified peptides are highlighted in shaded, boxed gray regions. Bold, red letters with a yellow square above them are identified Lysine sites of ubiquitination. B, ubiquitin lysine chain linkage of the FLNEDD4Lin vitro autoubiquitination assay was analyzed by LC/MS/MS. The peptide spectrum matches of each ubiquitin Lysine residue seen with a Gly-Gly modification is represented as a pie graph. C, representative MS/MS spectrum and sequence coverage of the peptide (TLSDYNIQkESTLHLVLR) containing a Gly-Gly modification on ubiquitin Lys63. Lower case k indicates Gly-Gly modification. D, in vitro ubiquitination assays of FLNEDD4L in the presence of UbWT, UbK48R, or UbK63R. Samples were quenched with reducing 2× SDS-PAGE loading buffer at 0, 10, 30, and 90 min and the gel was stained with colloidal Coomassie blue stain. The amount of unmodified FLNEDD4L protein, quantified by a densitometry analysis as a function of time, is shown as a percentage averaging all replicates. The average percentages ± SD for FLNEDD4L in the presence of Ub mutants are as follows (%): 100, 69 ± 2, 58 ± 10, 48 ± 14; 100, 87 ± 6, 60 ± 6, 40 ± 8; 100, 21 ± 5, 5 ± 3, 2 ± 1. All the assays were repeated at least twice (N ≥ 2). E, densitometry analysis, represented as a bar graph, of the average percentages of unmodified FLNEDD4L clustered by Ub mutant and taken at the indicated time points of (E). [file mmc4.pdf]

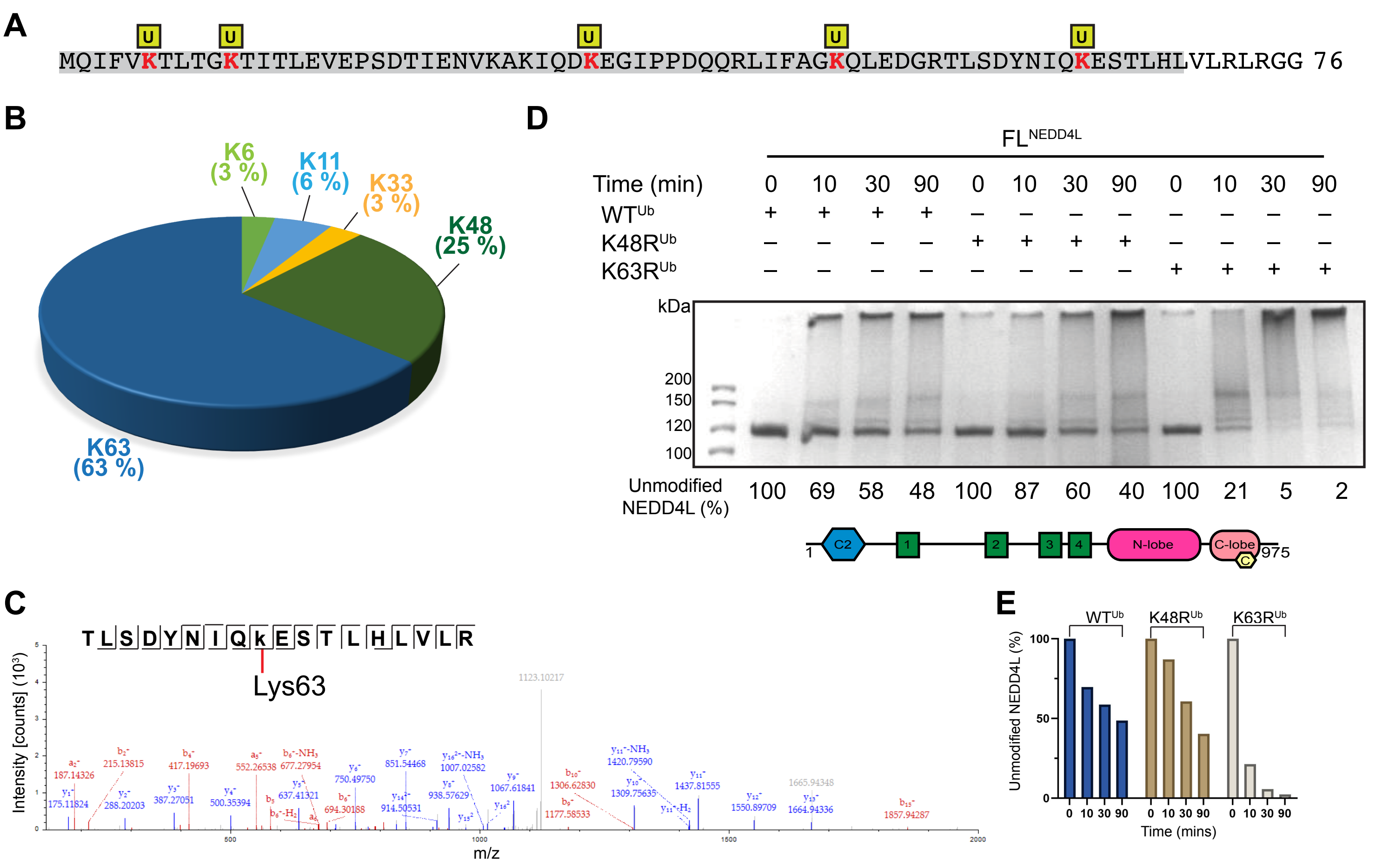
A
U
MQIFVKTLTGKTITLEVEPSDTIENVKAKIQDKEGIPPDQQRLIFAGKQLEDGRTLSDYNIQKESTLHLVLRLRGG 76
B
K6 (3 %)
K11 (6 %)
K33 (3 %)
K48 (25 %)
K63 (63 %)
C
TLSDYNIQkESTLHLVLR
Lys63
Intensity [counts] (10³)
m/z
1123.10217
D
FL NEDD4L
Time (min) 0 10 30 90 0 10 30 90 0 10 30 90
WTUb
K48RUb
K63RUb
kDa
200
150
120
100
Unmodified NEDD4L (%) 100 69 58 48 100 87 60 40 100 21 5 2
1
C2
1
2
3
4
N-lobe
C-lobe
C
975
E
WTUb
K48RUb
K63RUb
Unmodified NEDD4L (%)
100
50
0
0 10 30 90
Time (mins)
