# Supplementary material for: NEDD4L intramolecular interactions regulate its auto and substrate NaV1.5 ubiquitination
Source: J Biol Chem. 2024 Feb 2;300(3):105715. doi: 10.1016/j.jbc.2024.105715 (PMC10933555; doi:10.1016/j.jbc.2024.105715)
Supplement: Supporting Figure S6 — Ubiquitin chain linkage site-specifictiy of modified Nav1.5 shows primarily Lys63-linked chains.A, ubiquitin sequence coverage after di-Ub-NaV1.5DI-DII+PPSY band excision from the in vitro assay. Identified peptides are highlighted in shaded, boxed gray regions. Bold, red letters with a yellow square above them are identified Lysine sites of ubiquitination. B, ubiquitin lysine chain linkage of NaV1.5DI-DII+PPSY modified by FLNEDD4LLin vitro ubiquitination assay was analyzed by LC/MS/MS. The peptide spectrum matches of each ubiquitin Lysine residue seen with a Gly-Gly modification is represented as a pie graph. C, representative MS/MS spectrum and sequence coverage of the peptide (TLsDYNIQkESTLHLVLR) containing a Gly-Gly modification on ubiquitin Lys63 and (D) the peptide (LIFAGkQLEDGR) containing a Gly-Gly modification on ubiquitin Lys48. Lower case k indicates a Gly-Gly modification and lower case m indicates an oxidized methionine. E, fluorescent Western blot analysis of the in vitro FLNEDD4L autoubiquitination and NaV1.5DI-DII+PPSY substrate assays. Immunofluorescent staining was performed using an anti-K63-linked Ub (green) antibody. The purple arrow indicates unmodified NaV1.5DI-DII+PPSY and the pink arrow indicates FLNEDD4L as visible in (F) total protein stain of the same PVDF membrane. G, fluorescent Western blot analysis of the in vitro FLNEDD4L autoubiquitination and NaV1.5DI-DII+PPSY substrate assays. Immunofluorescent staining was performed using an anti-K48-linked Ub (green) antibody. The purple arrow indicates unmodified NaV1.5DI-DII+PPSY and the pink arrow indicates FLNEDD4L as visible in (H) total protein stain of the same PVDF membrane. [file mmc6.pdf]

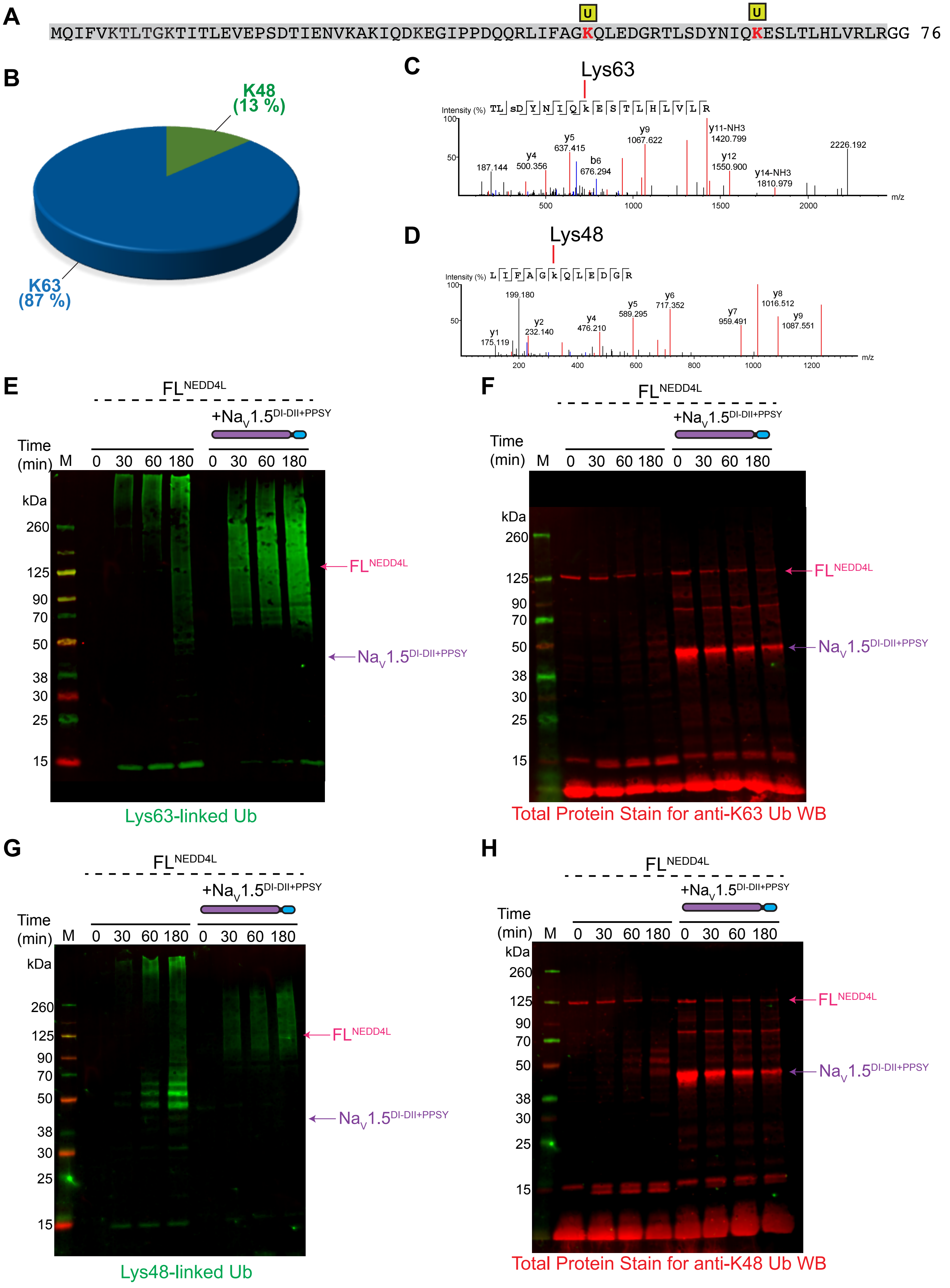

A
MQIFVKTLTGKTITLEVEPSDTIENVKAKIQDKEGIPPDQQRLIFAGKQLEDGRTLSDYNIQKESLTLHLVRLRGG 76
U
B
K48 (13 %)
K63 (87 %)
C
Lys63
Intensity (%)
TL sD Y N I Q k E S T L H L V L R
y5 637.415
y4 500.356
187.144
b6 676.294
y9 1067.622
y11-NH3 1420.799
y12 1550.900
y14-NH3 1810.979
2226.192
m/z
D
Lys48
Intensity (%)
L I F A G k Q L E D G R
199.180
y1 175.119
y2 232.140
y4 476.210
y5 589.295
y6 717.352
y7 959.491
y8 1016.512
y9 1087.551
m/z
E
F
G
H
FL^NEDD4L
+NaV1.5^DI-DII+PPSY
Time (min)
M
0 30 60 180 0 30 60 180
kDa
260
125
90
70
50
38
30
25
15
FL^NEDD4L
NaV1.5^DI-DII+PPSY
Lys63-linked Ub
Total Protein Stain for anti-K63 Ub WB
Lys48-linked Ub
Total Protein Stain for anti-K48 Ub WB
